# Noninvasive On-Skin Biosensors for Monitoring Diabetes Mellitus

**DOI:** 10.1007/s40820-025-01843-9

**Published:** 2025-07-31

**Authors:** Ali Sedighi, Tianyu Kou, Hui Huang, Yi Li

**Affiliations:** 1 Department of Materials, The University of Manchester, Oxford Road, Manchester, M13 9PL UK; 2 Singapore Institute of Manufacturing Technology (SIMTech), Agency for Science, Technology and Research (A*STAR), Singapore Institute of Technology, Singapore, Singapore

**Keywords:** Wearable biosensors, Multimodal sensors, Diabetes monitoring, Sweat biomarkers, Glucose biosensors

## Abstract

Diabetes mellitus represents a major global health issue, driving the need for noninvasive alternatives to traditional blood glucose monitoring methods. Recent advancements in wearable technology have introduced skin-interfaced biosensors capable of analyzing sweat and skin biomarkers, providing innovative solutions for diabetes diagnosis and monitoring. This review comprehensively discusses the current developments in noninvasive wearable biosensors, emphasizing simultaneous detection of biochemical biomarkers (such as glucose, cortisol, lactate, branched-chain amino acids, and cytokines) and physiological signals (including heart rate, blood pressure, and sweat rate) for accurate, personalized diabetes management. We explore innovations in multimodal sensor design, materials science, biorecognition elements, and integration techniques, highlighting the importance of advanced data analytics, artificial intelligence-driven predictive algorithms, and closed-loop therapeutic systems. Additionally, the review addresses ongoing challenges in biomarker validation, sensor stability, user compliance, data privacy, and regulatory considerations. A holistic, multimodal approach enabled by these next-generation wearable biosensors holds significant potential for improving patient outcomes and facilitating proactive healthcare interventions in diabetes management.

## Highlights

- A comprehensive and critical evaluation of recent advances in sweat-based biochemical and physiological biomarkers for noninvasive diabetes monitoring.
- A novel emphasis on multimodal sensor integration—combining biochemical and physiological signals—to enhance accuracy, contextual awareness, and reliability in real-time diabetes management.
- A forward-looking analysis of AI-driven biosensing systems, standardized protocols, and regulatory and ethical frameworks enabling autonomous, secure, and personalized diabetes care.

## Graphical abstract

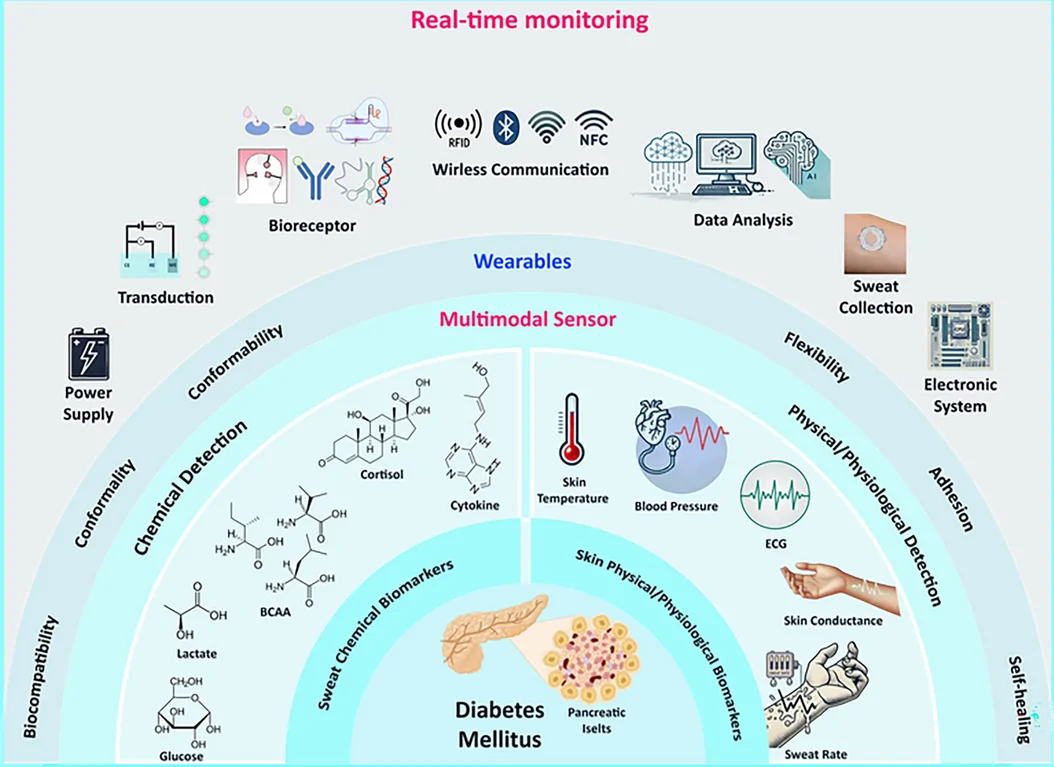

## Introduction

Diabetes mellitus (DM) is a chronic metabolic disease characterized by persistent hyperglycemia due to impaired insulin secretion, action, or both. With rising global prevalence and substantial economic impact, diabetes poses significant healthcare challenges requiring effective management strategies. Current diabetes management involves regular blood glucose monitoring, lifestyle modifications, and pharmacological interventions, including insulin therapy and oral hypoglycemic agents [1]. Conventional glucose monitoring using invasive methods, such as finger-pricking glucometers, presents limitations including discomfort, risk of infection, and poor patient compliance, highlighting the need for reliable noninvasive alternatives.

Recent advancements in wearable technologies offer promising approaches for noninvasive diabetes monitoring, leveraging biofluids accessible at the skin interface. Sweat, in particular, emerges as a convenient, easily collectible, and informative biofluid, reflecting various biochemical markers indicative of metabolic health. Notably, sweat glucose has demonstrated a consistent correlation with blood glucose concentrations, albeit typically at lower levels [2]. Beyond glucose, additional biochemical markers, such as branched-chain amino acids (BCAAs), cortisol, lactate, and cytokines, provide valuable insights into insulin resistance, metabolic stress, and diabetic complications. Monitoring these biomarkers through wearable sensors offers the potential for comprehensive, real-time metabolic assessment, significantly enhancing personalized disease management and patient outcomes.

Complementing chemical biomarker detection, physiological and biophysical parameters including sweat rate, heart rate, blood pressure, and skin conductivity further enhance health monitoring by indicating autonomic nervous system function, hydration status, and cardiovascular health [3]. For instance, alterations in sweat pH or skin conductivity can reflect conditions such as metabolic acidosis and autonomic neuropathy, common complications associated with uncontrolled diabetes [4, 5].

Eccrine sweat glands, distributed extensively across the body, facilitate the passive transport of smaller biochemical substances such as glucose and lactate from blood to sweat, making them ideal targets for wearable biosensor platforms. Sweat collection can be naturally induced via physical activity and heat exposure or actively stimulated using controlled iontophoresis. Effective sweat sampling techniques, including absorbent pads, wearable patches, and advanced microfluidic systems, are essential to ensure reliable sample integrity by minimizing contamination and evaporation.

Emerging wearable biosensors are increasingly adopting multiplexed sensing capabilities, allowing simultaneous detection of multiple biochemical and physiological signals. Advances in microfluidics, biocompatible and flexible materials, and sensor miniaturization have significantly enhanced the accuracy, comfort, and practicality of these devices. Additionally, biorecognition materials, such as enzymes, aptamers, molecularly imprinted polymers (MIPs), and nanozymes, have advanced specificity and sensitivity, enabling detection of trace-level biomarkers crucial for precise metabolic assessments.

For continuous health monitoring, the development of compact, ergonomic, and durable sensor platforms integrated with energy-efficient electronics is critical. Wearable biosensors must employ breathable, biocompatible materials that minimize skin irritation and maximize user comfort during prolonged wear. Flexible and conformable device designs that adapt to body contours without impeding daily activities are vital for ensuring user compliance.

Real-time data collection and transmission to smartphones or cloud-based platforms, coupled with advanced data analytics and artificial intelligence (AI) algorithms, are central to leveraging sensor data effectively. Machine learning techniques enable real-time analysis, predictive modeling, and personalized health insights, providing proactive intervention opportunities for diabetes management. Predictive algorithms analyzing biomarker variability and physiological data can anticipate critical glycemic events, optimizing therapeutic interventions tailored to individual physiological responses.

**Fig. 1:**
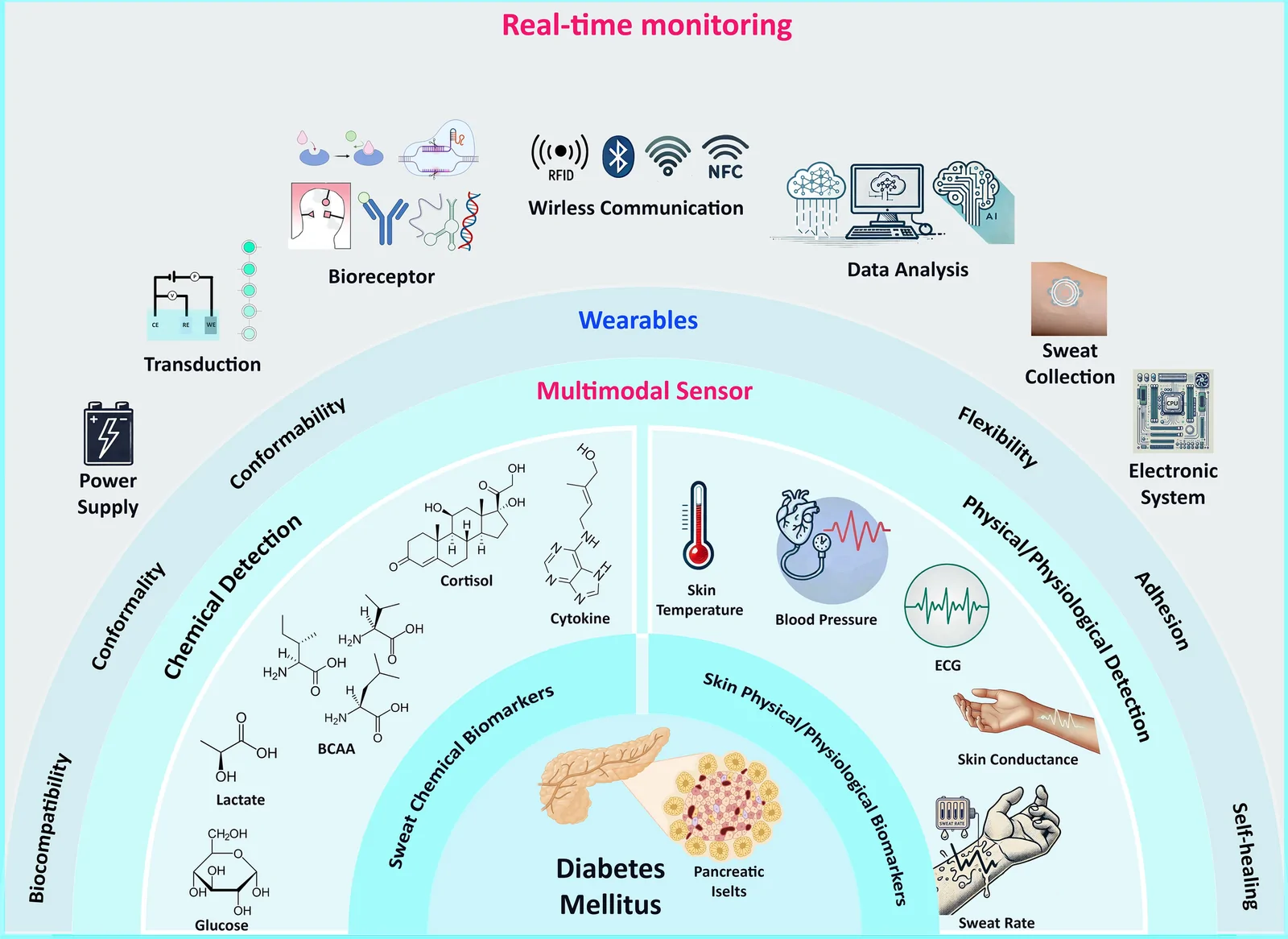
Overview of skin-interfaced wearable sensors for diabetes mellitus monitoring. Figures of diabetes mellitus and bioreceptor were created with Biorender.com

In contrast to prior reviews focusing solely on glucose detection, this review adopts a broader perspective by integrating chemical biomarkers, biophysical signals, and advanced wearable biosensor technologies, highlighting a comprehensive approach to diabetes monitoring. Emphasizing multimodal sensing, materials innovation, sensor validation, system integration, and AI-driven analytics, we provide a holistic view of current challenges and future directions in noninvasive on-skin diabetes biosensors (Fig. 1).

## Diabetes Mellitus: A Multifaceted Challenge

Diabetes mellitus is a complex metabolic disorder characterized by elevated blood glucose levels, or hyperglycemia. It is broadly categorized into two main types: Type 1 (T1DM) and Type 2 (T2DM). The global prevalence of DM continues to rise sharply, driven by urbanization, aging populations, and increasingly sedentary lifestyles. According to the International Diabetes Federation, approximately 540 million adults globally have diabetes, a number expected to reach 783 million by 2045. The World Health Organization (WHO) projects that DM may become the seventh leading cause of death worldwide by 2030. Alarmingly, a significant portion—22.8%—of adults with diabetes remain undiagnosed, as reported by the US Centers for Disease Control and Prevention (CDC). Diabetes not only severely impacts quality of life but also increases the risk of chronic diseases, including cardiovascular and chronic kidney disease, and is a leading cause of adult blindness. Currently, nearly half of diabetes cases remain undiagnosed, emphasizing the urgent need for improved diagnostic and monitoring tools [6–8].

**Fig. 2:**
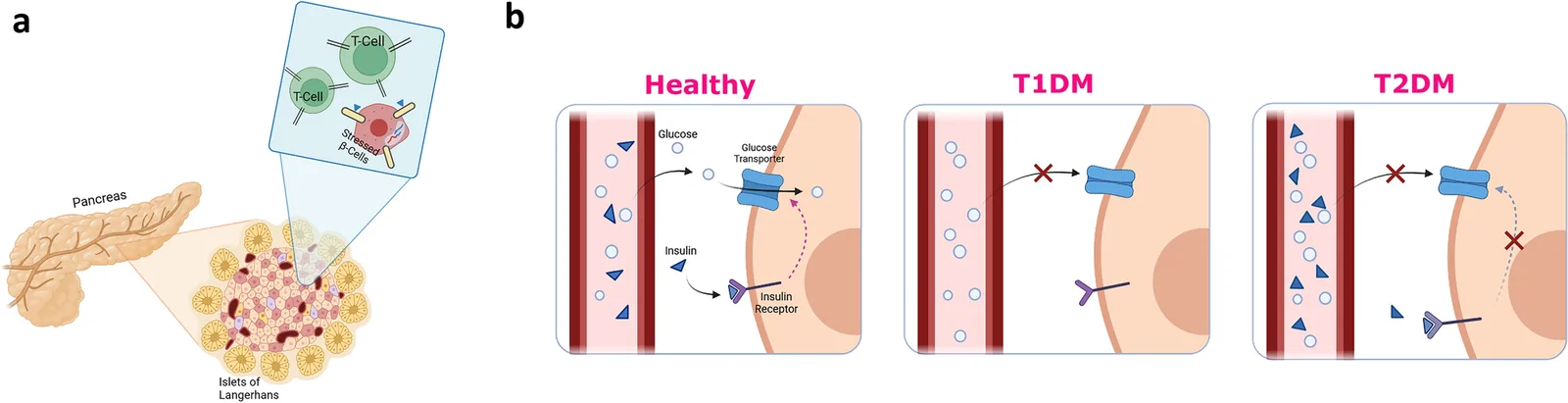
**a** T1DM schematic: T cells enter pancreatic islets via the bloodstream, stressing β cells due to high insulin demand or inflammation. **b** Comparison of normal physiology, T1DM, and T2DM: In a healthy body, insulin activates glucose transporters to clear blood glucose. In T1DM, β cells fail to produce insulin, preventing glucose removal. In T2DM, prolonged insulin overproduction desensitizes receptors, impairing glucose uptake. Created with BioRender.com

Type 1 diabetes mellitus (T1DM) results from autoimmune destruction of insulin-producing pancreatic β cells, typically driven by genetic predisposition, autoimmune factors, or viral infections [9]. This destruction causes a complete absence of insulin secretion. Globally, T1DM affects more than 34.2 million people, with around 11.7 million new diagnoses annually, including approximately 90,000 children [10]. T1DM is an autoimmune condition that eliminates insulin-producing β cells in the pancreatic islet (Fig. 2a). It leads to insulin deficiency and high blood sugar levels. In individuals without diabetes, pancreatic β cells within the islets of Langerhans dynamically secrete insulin to maintain stable glucose levels. In contrast, individuals with T1DM depend on exogenous insulin, delivered through injections or insulin pumps, to manage blood glucose levels and prevent severe acute and chronic complications. Achieving optimal insulin dosing that accurately matches metabolic needs is challenging, often resulting in fluctuating glucose levels and episodes of hyperglycemia or hypoglycemia [11].

Type 2 diabetes mellitus (T2DM), the most common form of diabetes, is characterized by insulin resistance, particularly in adipose tissue, skeletal muscle, and liver cells, alongside impaired insulin secretion from pancreatic β cells [12]. While genetics play a role in T2DM development, environmental and lifestyle factors, such as obesity, diet, physical inactivity, and aging, significantly influence disease onset [13]. Epigenetic changes, such as DNA methylation, have been identified in T2DM patients and are linked to lifestyle and environmental factors, increasing the risk of disease development. These epigenetic modifications can be environmentally triggered, inherited, or randomly acquired, collectively shaping the unique epigenetic signature of individual cells and tissues [13]. Such epigenetic markers show potential for predicting T2DM onset, vascular complication risks, and individual responses to therapeutic interventions and lifestyle changes, thereby paving the way for precision medicine. However, extensive research remains necessary before these biomarkers can be routinely implemented in clinical practice. Figure 2b summarizes key pathogenic mechanisms involved in both T1DM and T2DM.

Diabetes leads to extensive complications, broadly classified as macrovascular and microvascular (Table 1). Macrovascular complications, such as coronary artery and cerebrovascular diseases, are primary causes of diabetes-related mortality. Conversely, microvascular complications affecting the kidneys (nephropathy), eyes (retinopathy), and nerves (neuropathy) significantly contribute to morbidity and reduced quality of life [14]. With declining mortality from vascular diseases, diabetes is increasingly linked to heightened risks of cancer and dementia. Indeed, diabetes is associated with various malignancies [15], and increased susceptibility to infections, including COVID-19, pneumonia, and infections of the feet and kidneys, further elevating hospitalization and mortality risks [16]. Additionally, diabetes is frequently associated with cognitive impairment, functional disabilities, nonalcoholic fatty liver disease, obstructive sleep apnea, and depression [14]. Given the expanding spectrum of diabetes complications, comprehensive management strategies and regular screening protocols tailored to early detection and prevention are critical.

**Table 1:** Complications of DM

Complication	Symptoms	Risk Factors	Diagnostic Methods	Prevention Strategies	Prognosis	Incidence/Prevalence	References
Diabetic retinopathy	Vision loss, blurred vision	Poor blood sugar control, hypertension	Eye examination (retinal screening)	Blood sugar control, regular eye exams	It can lead to blindness if untreated	Highly prevalent in long-term diabetes	[17, 18]
Diabetic nephropathy	Swelling in limbs, urination changes	Poor blood sugar control, hypertension	Urine tests, blood tests	Blood sugar control, blood pressure management	Can progress to kidney failure	Common in long-term diabetes	[19, 20]
Diabetic neuropathy	Numbness and tingling in extremities	Poor blood sugar control	Physical exam, nerve function tests	Blood sugar control, foot care	It can lead to disability if severe	Very common in diabetes	[21]
Diabetic foot ulcer	Wounds on feet, infection	Poor blood circulation, neuropathy	Physical examination	Foot care, blood sugar control	Risk of infection, amputation	Common in individuals with neuropathy	[22, 23]
Cardiovascular disease	Chest pain, shortness of breath	Poor blood sugar control, obesity, hypertension	Blood tests, ECG, stress testing	Healthy diet, exercise, blood sugar control	Can be life-threatening	High risk in diabetics	[24, 25]
Stroke	Sudden numbness, confusion, trouble speaking	Hypertension, atrial fibrillation	CT scan, MRI	Blood pressure control, healthy lifestyle	Can lead to permanent disability or death	Increased risk for diabetics	[26, 27]
Peripheral artery disease	Pain in limbs, ulcers, hair loss on legs	Smoking, hypertension	Physical exam, ankle–brachial index (ABI) test	Quitting smoking, exercise	This can lead to amputation	Common in older adults with diabetes	[28, 29]
Diabetic osteoporosis	Bone pain, fractures	Poor blood sugar control, sedentary lifestyle	Bone density scanning (DEXA)	Exercise, Vitamin D, and calcium intake	Increased fracture risk	Higher prevalence in postmenopausal women	[30, 31]
Alzheimer	Memory loss, confusion	Poor blood sugar control, genetics	Neurological exam, brain imaging	Mental and physical activity, healthy diet	Progressive, no cure	Higher risk in diabetics	[32, 33]
Gum disease	Swollen, bleeding gums	Poor dental hygiene, smoking	Dental examinations	Regular dental care, quitting smoking	This can lead to tooth loss, infection	Common in diabetics	[34, 35]

### Diabetes Prevention and Management

Effective diabetes management requires a comprehensive, personalized approach, integrating diagnostic tools, lifestyle modifications, and pharmacological interventions. However, advancements in technology and health care have introduced innovative solutions that enhance patient compliance and overall quality of life. Early and accurate diagnosis is essential for managing diabetes, particularly type 2 diabetes mellitus (T2DM). Several large prospective studies have confirmed that identifying prediabetes—defined as impaired glucose tolerance and/or impaired fasting glucose—provides an opportunity to delay or prevent progression to T2DM [36]. Screening individuals at high risk of T2DM, using tools such as the Finnish Diabetes Risk Score or evaluating patients in cardiometabolic clinics (e.g., for metabolic syndrome, hypertension, or cardiovascular disease), often identifies individuals with HbA1c levels approaching 6.5% (48 mmol mol^−1^). Notably, prediabetes is variously defined as HbA1c levels of 5.7%–6.4% or 6.0%–6.4% (39–47 or 42–47 mmol mol^−1^, respectively) [37]. Traditional diagnostic methods, such as measuring plasma glucose levels, pose challenges due to pain, variability, and limited accessibility. In contrast, biosensors, particularly those incorporating nanotechnologies, provide a noninvasive, real-time alternative for glucose monitoring, leading to better disease management and treatment outcomes.

Weight management is crucial in controlling T2DM, as even a modest weight reduction of 5%–10% significantly improves glycemic control, triglyceride levels, HDL cholesterol concentrations, and blood pressure [38]. Structured weight management programs and bariatric surgery have been shown to induce diabetes remission, with effectiveness closely tied to the degree of weight loss [38]. Lifestyle interventions have also been shown to more than halve the rate of progression from prediabetes to T2DM across different age groups, as demonstrated in several studies and programs [39–41]. Encouragingly, implementation at the population level has yielded positive results; for instance, the UK National Health Service Diabetes Prevention Programme reported higher participation rates among older adults (including those aged ≥ 75 years), with superior reductions in body weight and HbA1c compared to younger individuals [42].

Conventional diabetes treatments, such as insulin injections for type 1 diabetes mellitus (T1DM) and oral medications for T2DM, remain fundamental. However, these treatments present challenges. Insulin injections can cause discomfort and adverse effects, while oral medications often require high dosages due to their short biological half-lives and potential side effects [43]. Among pharmacological options, metformin has demonstrated efficacy in younger individuals and those with obesity but has limited benefits in older adults with prediabetes [42]. Thiazolidinediones and acarbose have also been shown to reduce the progression from prediabetes to T2DM, including in older adults, though they are not currently approved for this indication [44].

**Fig. 3:**
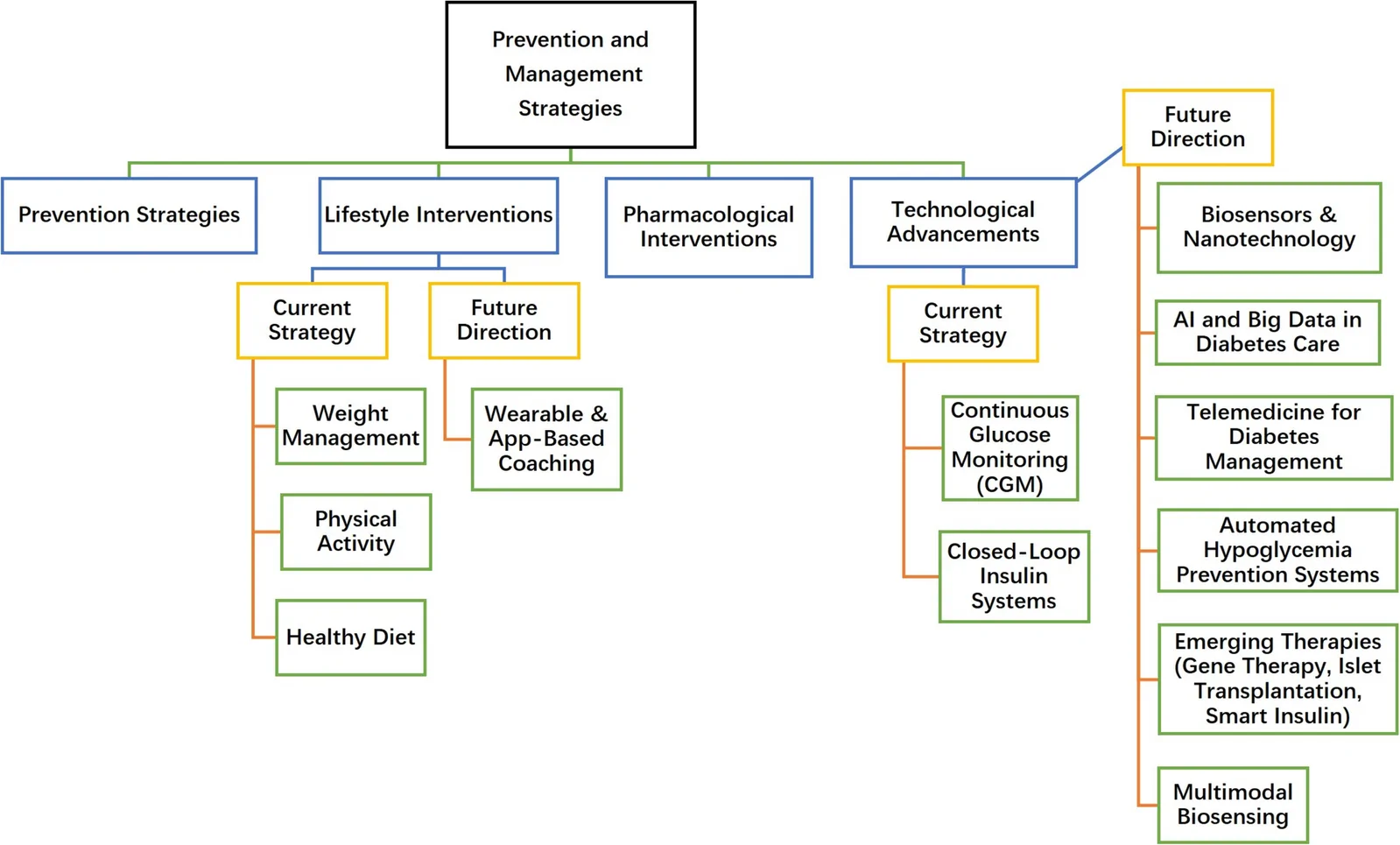
Comprehensive framework for diabetes prevention and management strategies, categorizing current strategies and future directions

A structured, multifaceted approach is required for the effective prevention and management of diabetes, integrating current strategies with emerging innovations in technology and pharmacology. Figure 3 shows the strategies and innovations aim to address the limitations of traditional diabetes management, improving patient compliance, disease monitoring, and treatment efficacy. By integrating AI, advanced biosensors, and precision medicine, the next generation of diabetes management will offer a more personalized, predictive, and preventative approach.

Technological innovations have significantly transformed diabetes care. Closed-loop devices, which integrate a glucose sensor with an insulin pump, allow for continuous glucose monitoring (CGM) and automated insulin delivery. One example is a fully integrated, wearable closed-loop system, combining a mesoporous microneedle-reverse iontophoretic glucose sensor with an iontophoretic insulin delivery component, enabling real-time glucose monitoring and insulin administration [45]. Point-of-care testing offers immediate results, enhancing diabetes management and adherence to self-care. CGM devices have been particularly effective in preventing hypoglycemia and improving glycemic control in both T1DM and T2DM.

Beyond standard treatments, nanomaterials infused with antidiabetic drugs have emerged as a promising strategy for controlled drug release, enhancing therapeutic efficacy, and minimizing side effects [46]. Gene therapy is also a compelling area of research, utilizing synthetic nucleic acids such as plasmid DNA, antisense RNA, microRNA, and small interfering RNA for pioneering diabetes treatments [47]. Additionally, islet transplantation remains a potential therapy but is hindered by limited donor availability, immune system rejection, and posttransplant complications [48].

Despite advancements in predictive, diagnostic, and prognostic biomarkers, blood glucose concentration and glycated hemoglobin (HbA1c) remain the gold standard for diabetes diagnosis and monitoring [49]. Given the importance of glycemic control in reducing microvascular and macrovascular complications, individualization of treatment—especially in older adults—is necessary. Guidelines now advocate less stringent HbA1c targets (e.g., < 8% [64 mmol mol^−1^] for older adults with long-standing diabetes and up to 8.5% [69 mmol mol^−1^] for frail individuals with complex needs) to reduce hypoglycemia risk while maintaining adequate symptom control [50].

A holistic approach to diabetes care in older adults extends beyond glucose control to managing cardiovascular disease risk factors, including blood pressure (target ≤ 140/85 mmHg, with lower targets for those with cardiovascular disease) and LDL cholesterol (goal < 2.6 or < 1.8 mmol L^−1^ for high-risk individuals) [51]. Personalized strategies, including tailored exercise programs (such as chair-based resistance training) and nutritional interventions, are essential for optimizing health outcomes in older adults.

## Diabetes Biomarkers

Diabetes biomarkers serve as essential tools for diagnosing, monitoring, and predicting disease progression. Traditionally, blood glucose and HbA1c levels have been the gold standard for diabetes detection. However, recent advances in metabolomics, proteomics, and genomics have led to the discovery of novel biomarkers that enhance diagnostic precision and allow for earlier intervention​. Among these emerging biomarkers, glycated albumin, fructosamine, and 1,5-anhydroglucitol (1,5-AHG) offer high specificity and sensitivity, particularly in cases where HbA1c measurements may not be feasible. In addition, metabolic markers, such as fetuin-A, branched-chain amino acids (BCAAs), adipokines, linoleoylglycylglycerophosphocholine (L-GPC), and lysophosphatidylcholine (LysoPC), have been identified through advanced metabolomic techniques, exhibiting strong correlations with blood glucose levels [52]​.

The gut microbiome also plays a role in diabetes development, with studies showing that alterations in gut microbiota composition influence glucose metabolism. Reduced Akkermansia muciniphila levels, for example, have been linked to impaired insulin secretion in individuals with newly diagnosed type 2 diabetes (T2DM)​ [53]. Additionally, gut-derived metabolites, such as imidazole propionate (ImP), bile acids (e.g., hyocholic acid), and phenolic acids, are associated with insulin resistance and glucose dysregulation [54].

Diabetic complications, including diabetic kidney disease (DKD) and diabetic retinopathy (DR), also have specific biomarker signatures. Some metabolites have shown promise as early indicators of these complications, offering opportunities for targeted intervention​. Moreover, genetic factors contribute to diabetes susceptibility, with over 30 T2DM-associated genetic variants identified [55]. However, these genetic predispositions alone provide limited predictive power, highlighting the need for multi-omics approaches that integrate genomic, proteomic, and metabolomic data​.

Innovative noninvasive glucose monitoring techniques, including salivary, sweat, and tear glucose analysis, are currently being explored, with some showing strong correlations to blood glucose levels. While these methods require further validation, they represent a promising avenue for future diabetes management​. The growing adoption of continuous glucose monitoring (CGM) systems and real-time biosensing technologies is further transforming diabetes care. These tools, when combined with artificial intelligence-driven analytics, provide personalized insights into glucose fluctuations, enabling earlier detection and improved glycemic control​.

### Biofluids

The introduction of noninvasive biomarkers has marked significant progress in healthcare compared to blood-based diagnostic tools. This development is significant for chronic diseases, where constant monitoring and timely intervention improve patient outcomes and prevent complications. Traditional invasive techniques can be burdensome, painful, and anxiety-inducing, especially for older people, infants, or those with needle phobia.

Noninvasive biomarkers provide an alternative, changing how we monitor health status and disease progression in comparison to blood (Table 2). Beyond less pain and distress, these biomarkers make sample collection safer, with less risk of infection or inflammation and could speed up diagnostic results. Moreover, continuous monitoring with wearable biosensors could completely change patient self-monitoring and disease management.

**Table 2:** Biofluids for biomarkers monitoring

Biofluid	Specifications	Target Biomarkers	Advantages	Disadvantages	References
Blood	Transports oxygen, nutrients, hormones, and waste products throughout the body	Metabolites, electrolytes, metals, proteins, peptides, amino acids, fatty acids, coenzymes, hormones, neurotransmitters, circulating RNAs, enzymes, vitamins, drugs	Easily accessible and can be collected in large quantities	Transports oxygen, nutrients, hormones, and waste products throughout the body	[56, 57]
Saliva	Secreted by salivary glands; aids in digestion, oral hygiene, and lubrication of the mouth	Hormones, enzymes, antibodies, metabolites, electrolytes, DNA and RNA molecules, proteins and peptides, inflammatory markers	Noninvasive and easy to collect	Secreted by salivary glands; aids in digestion, oral hygiene, and lubrication of the mouth	[58, 59]
Sweat	Produced by sweat glands; helps regulate body temperature through evaporation	Electrolytes, metabolites, proteins, peptides, amino acids, hormones, pH levels, drugs and drug metabolites, heavy metals	Noninvasive and can be collected continuously	Produced by sweat glands; helps regulate body temperature through evaporation	[60, 61]
Tears	Produced by lacrimal glands; keeps the eye surface moist, provides nutrients, and offers protection	Proteins, electrolytes, lipids, metabolites, inflammatory cytokines, growth factors, enzymes, microRNAs	Highly accessible	Produced by lacrimal glands; keeps the eye surface moist, provides nutrients, and offers protection	[62, 63]
Interstitial fluid (ISF)	Surrounds tissue cells, providing nutrients and removing waste. It is a filtrate of blood plasma but with lower protein content	Metabolites, electrolytes, metals, proteins, peptides, amino acids, fatty acids, coenzymes, hormones, neurotransmitters, circulating RNAs	Minimally invasive	Surrounds tissue cells, providing nutrients and removing waste. It is a filtrate of blood plasma but with lower protein content	[64, 65]
Breath	Contains volatile organic compounds (VOCs) and other gases that reflect metabolic processes in the body	Gaseous metabolites, inflammatory markers, bacteria, viruses	Noninvasive and painless	Contains volatile organic compounds (VOCs) and other gases that reflect metabolic processes in the body	[66, 67]

However, several challenges exist. Biofluids like sweat exhibit significant variations in salinity and pH, which can affect sensor accuracy and signal stability [68]. Additionally, these biofluids typically contain lower biomarker concentrations than blood and often exhibit a time lag in biomarker appearance relative to their blood levels. Furthermore, variability in biofluid production and evaporation rates complicates biomarker quantification, potentially leading to measurement inaccuracies.

### Noninvasive Skin Biomarkers

The skin is not only the largest organ of the human body but also the most accessible one for monitoring. Its thinness, mechanical properties, and easy accessibility make it ideal for noninvasive tracking of body motion, vital biosignals from the dermis and epidermis, as well as signals from internal organs, blood vessels, and muscles [69].

Metabolites present in sweat, sebum, and the products of protein degradation in the skin’s outer layers provide a rich source of information. These metabolites originate from eccrine and apocrine gland secretions, as well as sebum produced by sebaceous glands [70, 71]. ISF makes up a considerable volume of human skin and has a composition like plasma/serum but with generally lower concentrations of its components.

**Fig. 4:**
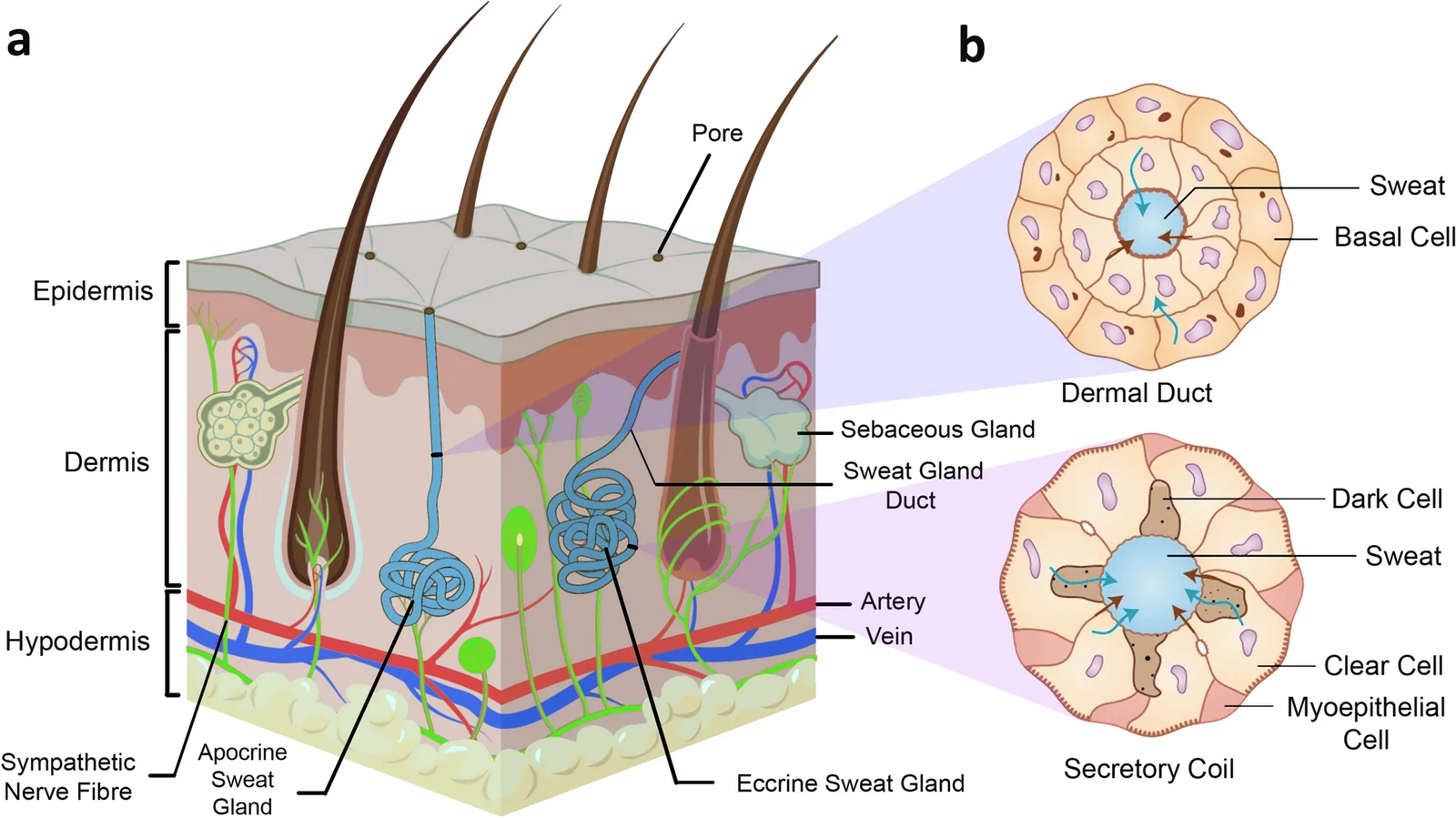
Physiology of skin and sweat secretion. **a** Schematic illustration of human skin structure. Created with Biorender.com. **b** Diagram of the dermal duct and secretory coil cross section. Reproduced with permission from Ref. [64]. Copyright 2019 Springer Nature

The skin is shown in three layers in Fig. 4a: the epidermis, dermis, and hypodermis. The epidermis, the outermost layer, acts as a protective barrier. Beneath it, the dermis contains various structures like hair follicles, sweat glands, and blood vessels. The deepest layer, the hypodermis, consists mainly of fat and connective tissue, providing insulation and cushioning to the body. In the dermis, hair follicles extend down from the epidermis and are associated with sebaceous glands, which secrete sebum to lubricate the skin and hair [72]. The sweat glands, which play a critical role in thermoregulation and waste excretion, are also in the dermis.

**Sweat**

Sweat is complex and easily collected. Thus, it is becoming a focal point for wearable biosensor technology. It offers a less invasive alternative to blood tests, allowing ongoing health checks. Sweat is a biofluid that contains potential biomarkers, including glucose, lactate, cortisol, sodium, potassium, and chloride [73]. Studying these biomarkers in sweat provides insights into various health conditions, including DM.

Sweat plays a vital role in body heat regulation. In adults under 65, the body’s sweat production is reliable, making it a dependable source for testing. However, in infants, sweat production is lower due to their underdeveloped thermoregulation, leading them to rely on other mechanisms such as increased dry heat loss and higher skin temperatures. Additionally, as people age, the production and distribution of sweat glands decrease, which can affect thermoregulation and sweat production [61].

Eccrine sweat glands, spread throughout the skin, regulate body temperature by secreting sweat directly onto the skin’s surface. Apocrine sweat glands in specific areas like the armpits and groin are larger, and their ducts open into hair follicles [74]. These glands secrete a thicker fluid, leading to body odor when broken down by bacteria.

Figure 4b details the structure of a sweat gland, focusing on the secretory coil and the ducts. The secretory coil, where sweat production begins, is composed of dark cells, clear cells, and myoepithelial cells. Dark cells contribute to sweat production, while clear cells mainly secrete water and electrolytes. Myoepithelial cells surround the secretory cells and contract to help expel the sweat from the gland into the duct [74]. The sweat then moves through the dermal duct toward the skin surface or into a hair follicle in the case of apocrine glands. Basal cells line the ducts and play a role in maintaining the glandular structure [75].

Sweat sampling is crucial part of sweat monitoring. Iontophoresis—applying a mild electric current to deliver cholinergic agents like pilocarpine or carbachol—has emerged as a promising approach for generating sweat in sedentary or clinical settings. While pilocarpine is widely used, carbachol-based induction offers extended and more stable sweat production, facilitating continuous biomarker monitoring during both rest and activity [76]. However, discrepancies in electrolyte concentrations, pH, and molecular profiles across induction methods raise questions about data comparability. For example, iontophoretically induced sweat often exhibits elevated pH and reduced bicarbonate reabsorption, which can affect sensor calibration and reliability [60, 77].

In parallel, innovations in skin-conformal microfluidic systems are improving sweat collection efficiency and mitigating interpersonal variability. Recent designs employ capillary bursting valves, passive Tesla valves, and wedge-shaped wettability gradients to regulate directional flow and enable time-resolved sweat sampling, minimizing the mixing of old and new sweat [78–81]. Janus textiles and porous hydrogels have also been integrated into microfluidic networks to passively draw sweat while ensuring consistent flow across variable secretion rates and skin types [82, 83]. These fluid-handling strategies are particularly promising for enhancing sensor reliability across diverse user populations without requiring active pumping systems.

Sweat-based health monitoring presents several significant challenges. One of the primary issues is the lack of comprehensive data establishing a precise relationship between sweat and blood levels of key biomarkers such as glucose, lactate, and electrolytes [84]. Additionally, the mechanisms governing biomarker transport into sweat remain poorly understood. While water and small lipophilic molecules (e.g., steroids) diffuse quickly through sweat gland membranes, larger or charged biomarkers like glucose must navigate the extracellular matrix, a process influenced by sweat gland activity and matrix composition [68]. Another challenge lies in sweat sampling for both research and diagnostic applications. Chemical stimulants such as pilocarpine and carbachol are commonly used to induce sweating. However, it remains unclear whether stimulated sweat is compositionally comparable to sweat produced naturally through heat or exercise, raising concerns about the accuracy and reproducibility of biomarker measurements [68].

#### Chemical Biomarkers

Several biomarkers in sweat, including glucose, lactate, cortisol, cytokines, and branched-chained amino acids (BCAAs), play crucial roles in DM management (Table 3). These biomarkers can serve as noninvasive checkpoints to monitor disease progression and treatment outcomes. Research indicates a significant correlation between biomarkers in sweat and blood. However, some challenges remain. One key issue is the lag time between blood and sweat measurements, ranging from a few to tens of minutes [85]. This lag is minimized by the highly vascularized nature of the secretory coil, which allows for rapid circulation of blood changes [64]. In the case of transcellular transport, the rate of diffusion determines how quickly analytes enter the lumen of the sweat duct. Once inside the lumen, osmotic fluid flow (sweat rate) drives advective transport, dictating the time it takes for the analyte to move from secretion to elution [86].

**Table 3:** Noninvasive sweat-based biomarkers correlated to DM

Biomarker	Molecular weight (g/mol)	Concentration in blood (mM)	Concentration in sweat (mM)	Sensing transduction	Sampling method	Correlation to diabetes	Referencess
Lactate	90	0.5–25	5–60	Electrochemical (amperometric, enzymatic sensors), colorimetric (enzymatic assays)	Passive absorption (textile patches, absorbent pads), microfluidic collection	Lactate serves as an early biomarker for T2DM, with levels reflecting changes in energy metabolism and insulin resistance, particularly during physical activity	[87–91]
Glucose	180	3.3–17.3	0.01–0.3	Electrochemical (enzymatic amperometric sensors, MIP-based sensors), optical (fluorescence-based assays)	Passive absorption (hydrogel patches, wearable microfluidics), Iontophoresis (active stimulation)	Blood glucose levels are primary markers for diabetes. While there is a correlation between sweat and blood glucose levels, this relationship is influenced by factors such as sweat rate, hydration status, and electrolyte balance	[60, 92–95]
BCAAs	117–131	0.2–1.2	0.2–1	Electrochemical (amperometric sensors, MIPs), optical (fluorescence-based assays)	Passive absorption (wearable textile patches, microfluidics), iontophoresis (pilocarpine-induced sweating)	Elevated BCAA levels in blood are strongly associated with insulin resistance, T2DM, and metabolic disorders. While there is a correlation between serum and sweat BCAA levels, localized metabolism in sweat glands may influence their concentrations	[60, 76, 96, 97]
Cytokines (interleukin-6)	21,000	2.4–5.6 × 10^–10^	3.7–6.9 × 10^–10^	Electrochemical (aptamer-based impedimetric sensors, immunosensors), optical (surface plasmon resonance, SPR)	Passive absorption (hydrogel-based patches), microfluidic sampling	Pro-inflammatory cytokines, such as IL-6 and TNF-α, play a role in insulin resistance and beta-cell dysfunction in diabetes, as part of the body's inflammatory response to metabolic dysregulation	[60, 98, 99]
Cortisol	362	0.07–690 × 10^–3^	0.1–20 × 10^–3^	Electrochemical (immunosensors, MIPs), optical (colorimetric, fluorescence assays)	Passive absorption (textile patches, hydrogel patches), microfluidic sampling	Cortisol promotes gluconeogenesis and inhibits glucose uptake. Persistently elevated cortisol levels are associated with insulin resistance and metabolic disturbances	[100–103]

**Glucose**

Blood glucose levels are the primary marker for diabetes, directly influenced by the body’s ability to produce and utilize insulin. Carbohydrate digestion breaks macronutrients into glucose, which is absorbed into the bloodstream, raising blood sugar levels [10]. In a healthy system, the pancreas detects this rise and secretes insulin, facilitating cellular glucose uptake [104]. Once inside the cell, glucose is either used for energy or stored as glycogen in the liver and muscles [105]. In T1DM, an autoimmune response leads to insulin deficiency, preventing glucose entry into cells and causing hyperglycemia [106]. In T2DM, cells develop insulin resistance, or the pancreas produces insufficient insulin, leading to inefficient glucose uptake and elevated blood sugar levels [107]. Chronic hyperglycemia can damage blood vessels and nerves, increasing the risk of severe complications [108, 109].

Glucose monitoring is essential for diabetes management, and sweat-based glucose sensors offer a promising noninvasive alternative. Research indicates that sweat glucose levels correlate with blood glucose, though this relationship is influenced by skin permeability, sweat rate, and local blood flow [110, 111]. Due to its large molecular size and polarity, glucose passes through sweat gland junctions at much lower concentrations—roughly 100 times lower than in blood [60]. A touch-based sweat glucose sensor demonstrated a Pearson correlation coefficient of 0.77 between sweat and blood glucose, improving to 0.95 after applying personalized calibration for sweat rate and skin properties [112]. Similarly, studies have reported a positive correlation (*r* = 0.75) between blood and noninvasively collected sweat glucose [113]. However, some studies found no significant correlation between glucose levels in iontophoretic and exercise-stimulated sweat, suggesting that temperature, pH, sweat rate, and individual differences affect accuracy [114–116].

**Lactate**

Lactate is a by-product of anaerobic metabolism, primarily associated with muscle activity but also produced in other tissues, including the skin [117]. Under low oxygen conditions, such as during intense exercise, cells shift to anaerobic metabolism, increasing lactate production [118]. In diabetes, poor glucose regulation can disrupt normal metabolism, altering the balance between aerobic and anaerobic pathways, which affects lactate levels [119].

Insulin regulates both glucose and lactate metabolism. In diabetes, impaired insulin function can cause abnormally high lactate levels, especially after a glucose load, highlighting altered metabolic processes [120]. Fasting plasma lactate levels may also be elevated due to insulin resistance or deficiency, serving as a potential metabolic marker for diabetes [121].

Several studies support the link between blood lactate and diabetes, particularly T2DM. One study found that postmenopausal women with T2DM had higher basal and exercise-induced blood lactate levels compared to obese controls, with a positive correlation between lactate and HbA1c, indicating a connection between chronic hyperglycemia and elevated lactate [122]. Another study measuring lactate and glycerol levels during oral glucose tolerance tests and hyperinsulinemic–euglycemic clamps suggested that rising plasma lactate levels could be an early marker of insulin resistance and T2DM development [123]. In T1DM, studies have linked elevated plasma lactate levels to glycogenic hepatopathy, a condition characterized by excess glycogen accumulation in the liver. Poor glycemic control was associated with increased lactate levels, but these metabolic abnormalities improved with better glucose regulation, reinforcing the importance of glycemic control over rare metabolic disorders [90].

Lactate can be measured in sweat, offering a noninvasive alternative for monitoring metabolic status. Research has shown that sweat lactate levels rise in parallel with blood lactate levels during exercise, with strong correlations (*r* > 0.8 in active muscles and *r* = 0.7 in latent muscles) [124]. Notably, sweat lactate responds more rapidly than blood lactate, making it valuable for sports medicine and athlete monitoring.

While sweat lactate shows promise as a diabetes biomarker, more research is needed to assess its reliability and limitations. Factors, such as hydration, exercise intensity, and ambient temperature, can influence sweat composition, potentially affecting measurement accuracy [125, 126]. While sweat lactate shows promise as a diabetes biomarker, more research is needed to assess its reliability and limitations. Factors, such as hydration, exercise intensity, and ambient temperature, can influence sweat composition, potentially affecting measurement accuracy.

**Branched-Chain Amino Acids**

Branched-chain amino acids (BCAAs)—leucine, isoleucine, and valine—have emerged as potential biomarkers for T2DM due to their elevated circulating levels in individuals with obesity and insulin resistance [60, 127]. Although hyperglycemia and dyslipidemia are defining features of diabetes, amino acid imbalances, particularly involving BCAAs, are increasingly implicated in its pathogenesis [128, 129].

Multiple clinical studies have reported that high blood BCAA levels positively correlate with insulin resistance and HbA1c, with some evidence suggesting they may even predict future development of T2DM [76]. One proposed mechanism is that chronically elevated BCAA levels activate the mammalian target of rapamycin complex 1 (mTORC1) signaling pathway, which disrupts insulin receptor signaling and contributes to insulin resistance [130]. Another hypothesis suggests that abnormal BCAA metabolism leads to the accumulation of metabolic intermediates, which may induce mitochondrial dysfunction and stress responses associated with insulin resistance and T2DM progression [131].

In individuals with T2DM, elevated BCAA levels are also linked to reduced mitochondrial oxidative capacity and impaired metabolic flexibility, further suggesting that inefficient BCAA oxidation contributes to metabolic disturbances [132]. Importantly, these associations are observed independently of body mass index (BMI), highlighting a direct role of BCAAs in metabolic disease risk [133]. BCAA concentrations also show strong correlations with insulin-related markers, including C-peptide, insulin, and HOMA-IR, reinforcing their relevance to insulin function. Moreover, long-term dietary BCAA intake has been associated with an increased risk of developing T2DM, independent of conventional risk factors such as BMI [134].

Emerging research supports the use of sweat BCAA levels as a noninvasive indicator of metabolic status. Elevated BCAAs in sweat have been associated with T2DM, obesity, insulin resistance, and cardiovascular disease [76, 129]. A positive correlation between serum and sweat BCAA concentrations has been observed, particularly in individuals with metabolic dysfunction [76]. Additionally, postprandial studies have shown that sweat leucine and total BCAA levels fluctuate according to metabolic condition, with protein-rich meals and BCAA supplementation leading to increases in both blood glucose and insulin levels. These findings suggest that sweat-based BCAA analysis may offer valuable insights into metabolic health and insulin dynamics in a noninvasive manner.

**Cytokines**

Cytokines are small signaling proteins secreted primarily by immune cells that play a central role in cell communication and immune responses [135]. Both type 1 and type 2 diabetes are characterized by chronic low-grade inflammation, driven by factors such as hyperglycemia, insulin resistance, and autoimmune destruction of pancreatic beta cells [136]. In response, the body releases pro-inflammatory cytokines, notably interleukin-6 (IL-6) and tumor necrosis factor-alpha (TNF-α), which have been closely linked to insulin resistance and beta-cell dysfunction [137]. Elevated serum IL-6 levels have been reported in T1DM patients, with variability influenced by age, disease duration, and ethnicity [138]. Studies in animal models, such as nonobese diabetic mice and biobreeding rats, show that both pro-inflammatory (e.g., IL-1, TNF-α, IFN-α) and type 1 cytokines (e.g., IFN-γ, IL-2) contribute to beta-cell destruction and the development of T1DM [139, 140].

A large cohort study (*n* = 6085) found that IL-6 and TNF-α levels positively correlated with fasting plasma glucose and insulin levels, with significantly higher levels in diabetic individuals—even after adjusting for confounding factors [141]. These findings support the strong association between elevated cytokines and diabetes.

In diabetes, systemic inflammation driven by metabolic dysregulation can extend to the skin, leading to cytokine excretion through sweat glands [142]. Multiple studies have demonstrated a strong correlation between sweat and plasma cytokine levels [98, 143]. Marques-Deak et al. showed that cytokines, such as IL-1α, IL-1β, IL-6, TNF-α, IL-8, and TGF-β, are present in sweat at levels comparable to plasma, suggesting sweat may reflect systemic inflammatory status [143, 144]. While sweat cytokines hold promise as noninvasive biomarkers for diabetes-related inflammation, further studies are needed to clarify their mechanisms and establish reliable diagnostic applications.

**Cortisol**

Cortisol, produced by the adrenal glands, plays a key role in stress response, immune regulation, inflammation, and glucose metabolism [145]. It promotes gluconeogenesis—the generation of glucose from noncarbohydrate sources—particularly during stress or fasting, while simultaneously inhibiting glucose uptake in muscle and fat tissues, thereby raising blood glucose levels [114, 145]. Chronic elevation of cortisol is associated with insulin resistance, a hallmark of T2DM [146, 147]. Studies have also underscored cortisol’s pathogenic role in metabolic syndrome, which is closely linked to T2DM [102].

The presence of chronic complications in T2DM, such as macroangiopathy, retinopathy, and neuropathy, correlates with hypothalamic–pituitary–adrenal (HPA) axis activity [117]. In a study of 170 T2DM patients and 71 matched controls, hypothalamic–pituitary–adrenal (HPA) axis activity was higher in diabetic individuals, especially those with complications like retinopathy, neuropathy, and macroangiopathy [102]. Cortisol levels were positively correlated with the number and severity of these complications [117].

Cortisol is also implicated in the impact of psychological stress on glucose metabolism. The "fight or flight" response elevates stress hormones, including cortisol and adrenaline, which increase gluconeogenesis and reduce insulin sensitivity, raising blood glucose [148, 149]. In people with diabetes, stress can exacerbate hyperglycemia, creating a harmful feedback loop. Prolonged stress leads to allostatic load, contributing to insulin resistance and low-grade inflammation, both of which promote T2DM development [150]. Epidemiological studies also associate chronic stress, depression, and early life adversity with increased diabetes risk [122, 150].

Cortisol is detectable in sweat, making it a promising candidate for noninvasive monitoring of metabolic and stress-related states [151]. Studies have found that individuals with T2DM tend to have higher cortisol levels in sweat, blood, saliva, and urine compared to healthy individuals [10, 102]. One study found a significant correlation between sweat and salivary cortisol levels (*r*^2^ = 0.30, *P* < 0.05), suggesting that sweat may reflect systemic cortisol activity [152]. However, this relationship is influenced by factors like exercise, diet, and individual variability, and further research is needed to validate its utility in diabetes monitoring [151].

#### Physiological/Physical Biomarkers

**Fig. 5:**
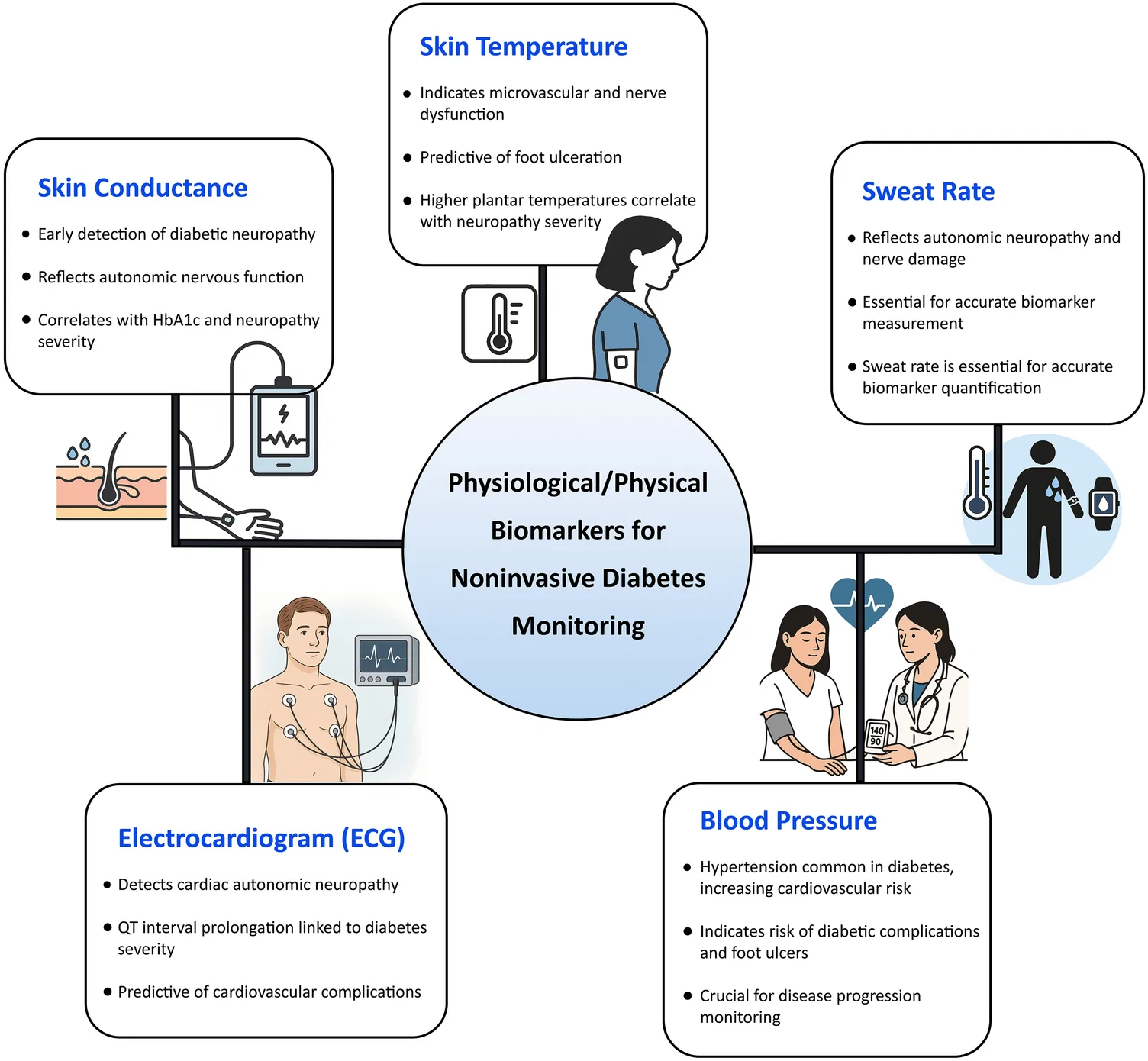
Overview of key physiological and physical biomarkers relevant to noninvasive wearable monitoring for diabetes mellitus management. Each biomarker provides complementary insights into autonomic nervous system function, cardiovascular health, and disease progression, collectively enabling personalized diabetes care

Figure 5 effectively summarizes the critical elements discussed in the section, showing that physiological and physical biomarkers contribute significantly to noninvasive diabetes monitoring.

##### Skin Conductance

Electrochemical skin conductance (ESC), also known as galvanic skin response (GSR), is emerging as a valuable physical biomarker for assessing autonomic nervous system function and diabetic neuropathy [153]. ESC reflects sweat gland activity, which is often impaired by chronic hyperglycemia. Because the autonomic nervous system regulates sweat secretion, changes in ESC can serve as early indicators of autonomic dysfunction in diabetes [154]. Numerous studies have shown reduced ESC in individuals with diabetic peripheral neuropathy, cardiac autonomic neuropathy, and metabolic syndrome, compared to healthy controls [155–157]. Lower ESC has also been correlated with higher HbA1c, elevated C-reactive protein, and lower ankle–brachial index, suggesting its value in tracking small fiber neuropathy severity [155, 156]. In a multi-ethnic study, ESC was strongly associated with diabetic kidney disease, indicating its potential for noninvasive early detection in T2DM patients [158]. However, another study using meal tolerance tests found that although ESC variability helped classify diabetic patients, its overall accuracy as a diabetes screening tool was limited [159].

In the context of cardiac autonomic neuropathy, a study of Chinese patients found ESC to be a feasible, noninvasive screening tool, with results comparable to cardiovascular reflex tests—the clinical gold standard [160]. Similarly, combining ESC with heart rate variability (HRV) improved risk stratification in T2DM patients. Those with lower ESC and reduced HRV, particularly in foot measurements and standard deviation of the normal-to-normal intervals values, exhibited greater cardiovascular autonomic impairment [161].

Based on these findings, researchers propose a stratified approach using ESC to screen and monitor cardiovascular autonomic neuropathy, where lower ESC readings indicate the need for closer clinical follow-up [162]. As a quick and noninvasive measure, ESC holds promise for detecting and tracking diabetic complications, especially in autonomic function.

##### Skin Temperature

Prolonged hyperglycemia in diabetes affects both nerves and blood vessels, leading to skin changes that may serve as early indicators of complications and signal the need for adjustments in management. Damage to microvascular and macrovascular systems impairs blood flow, especially in the hands and feet [163, 164], while diabetic neuropathy disrupts autonomic functions such as sweating and thermoregulation, often resulting in abnormal skin temperatures [3, 165].

Although not yet a clinical standard, skin temperature monitoring has drawn interest as a noninvasive tool for assessing vascular and nerve function. A study by Kitaoka et al. found delayed temperature recovery after cold exposure in diabetic patients, particularly those with severe peripheral neuropathy, linking skin temperature regulation to sympathetic nerve dysfunction [166]. Another study showed that diabetic patients had reduced skin blood flow and a correlation between skin temperature and blood flow under different humidity levels, emphasizing diabetes’ impact on vascular responses [167].

Diabetes mellitus also affects foot skin temperature. Infrared thermography has been applied to assess foot temperature, with Bagavathiappan et al. reporting higher plantar temperatures (32–35 °C) in neuropathic T2DM patients compared to non-neuropathic ones (27–30 °C) [168]. Additionally, a systematic review and meta-analysis of studies from 1960 to July 2011 identified an increase in skin temperature at the same site on contralateral limbs as an early predictor of foot ulceration [169]. Van Netten et al. identified a 2.2 °C difference as an early indicator (76% sensitivity, 40% specificity), and a 1.35 °C average temperature gap between feet provided 89% sensitivity and 78% specificity for detecting complications [170].

Diabetes also raises infection and inflammation risk. High glucose impairs immune function [171], while poor circulation and nerve damage increase the likelihood of chronic wounds, especially in the feet [172, 173]. Infections trigger local temperature rises due to inflammation, increased blood flow, and immune cell activity [174].

However, skin temperature monitoring has limitations. It may not distinguish diabetic complications from other causes of temperature changes, and its accuracy can be affected by ambient conditions and patient activity [170, 175]. Relying solely on skin temperature may delay diagnosis, as changes often appear after complications arise. Therefore, it is best used as a complementary tool alongside other diagnostic methods and clinical assessments.

##### Sweat Rate

Altered sweat production is a key indicator of autonomic dysfunction and useful for early detection and monitoring of diabetic neuropathy. Diabetes disrupts sweat gland activity, affecting both sweat rate and composition, which can serve as noninvasive biomarkers for blood glucose levels [176]. Reduced sweat gland innervation leads to decreased sweat output, with severity increasing alongside neuropathy progression. This is supported by a reduction in sweat duct cross-sectional area and corneal nerve fiber density [176].​​ Depending on the extent of nerve damage, patients may experience anhidrosis (reduced sweating) or hyperhidrosis (excessive sweating) [177].

Monitoring changes in sweat rate can reveal neuropathy progression. Both directly stimulated and axon reflex-mediated sudomotor responses offer valuable insights into peripheral autonomic nerve function [178]. Diabetic patients show significantly lower sweat output per gland and skin area, along with reduced sweat gland density, particularly in the lower leg—contributing to complications like diabetic foot [179].

Kennedy et al. found that sweating deficiency correlated with clinical pain perception, though not with alpha motor conduction velocity or muscle denervation [180]. Another study showed that diabetic patients had significantly altered sympathetic sweat responses and skin vasomotor reflex amplitudes compared to controls, highlighting autonomic dysfunction [181]. These assessments help evaluate autonomic involvement in diabetes. However, factors, like environmental conditions, individual variability, and comorbidities, can affect sweat production, complicating interpretation. The duration and severity of diabetes also influence autonomic function, underscoring the need to consider patient history.

Sweat rate is essential for accurate biomarker quantification. Without it, analyte concentrations can be misleading due to dilution effects. Knowing sweat secretion rate enables calculation of analyte flux. In low-volume sweat scenarios, correcting for transepidermal water loss is also crucial to ensure measurement accuracy [116].

##### Electrocardiogram

Diabetes can alter the heart’s electrical activity due to its impact on the cardiovascular system. These changes may be detected through ECG, offering a noninvasive method to identify individuals at risk or already affected. ECG plays a vital role in assessing cardiovascular risks linked to diabetes. Stern and Sclarowsky highlighted diabetes-related ECG changes and supported its use for early detection, monitoring, and evaluating cardiac autonomic neuropathy and silent myocardial ischemia [182]. One study emphasized abnormal ECG findings—alongside age, heart failure history, CRP levels, and urinary protein/creatinine ratio—as predictors of cardiovascular risk, underscoring the link between diabetes, CKD, and heart health [183]. Another study found a strong correlation between prolonged QT interval dispersion and complications like myocardial ischemia, left ventricular hypertrophy, and autonomic dysfunction in diabetic patients [184]. QT dispersion proved especially useful for predicting ischemia and left ventricular hypertrophy—major contributors to morbidity and mortality in diabetes.

Detailed ECG analysis, including QT interval prolongation and resting tachycardia, can also predict complications such as cardiac autonomic neuropathy, nephropathy, and retinopathy [185]. QT prolongation showed reasonable sensitivity and specificity for diagnosing cardiac autonomic neuropathy, with its severity correlating with the extent of nerve damage.

Although ECG abnormalities and heart rate variability are not direct biomarkers for diabetes, they are valuable indicators of cardiovascular complications. Monitoring these changes can support early intervention and improved risk management in diabetic patients.

##### Blood Pressure

Diabetes significantly increases the risk of cardiovascular disease, with hypertension serving as a major contributing factor [186]. Hypertension, or high blood pressure, arises from increased arterial resistance, forcing the heart to work harder to circulate blood. Individuals with diabetes are twice as likely to develop hypertension compared with nondiabetic individuals [187]. If left untreated, hypertension markedly raises the likelihood of heart disease and stroke [188]. Among people with diabetes, elevated blood pressure further heightens the risk of heart disease. Approximately two-thirds of adults with diabetes present blood pressure readings above 140/90 mmHg or require antihypertensive medications, thereby increasing their vulnerability to additional diseases and mortality, especially if diabetic nephropathy is also present [189]. In diabetes, hypertension leads to a more than sevenfold increase in the risk of death, whereas in individuals who have both diabetes and diabetic nephropathy, this risk soars to 37 times higher compared to those without these conditions [188, 190, 191].

Insulin resistance, common in diabetes, is linked to hypertension, vascular stiffness, and impaired vasodilation [192, 193]. It may also disrupt nitric oxide signaling, increase sympathetic activity, and activate the renin–angiotensin–aldosterone system, highlighting the interconnected nature of hyperglycemia and high blood pressure [194, 195].

Diabetes also increases foot complication risks due to poor circulation, peripheral arterial disease, and neuropathy [29, 196, 197], which impair blood flow and sensation, leading to infections, ulcers, and delayed wound healing [198, 199]. Proper diabetes management, routine foot checks, and appropriate footwear are essential to prevent severe complications and preserve foot health.

Continuously monitoring blood pressure in diabetic patients is crucial for forecasting disease progression and potential complications. Studies indicate that home blood pressure telemonitoring—combined with automated self-care messages—significantly decreases daytime systolic blood pressure and improves the likelihood of achieving target pressure levels without additional clinic visits or medication [200]. Blood pressure readings are also vital in predicting outcomes for diabetic foot ulcers: higher ankle and toe pressures correlate with improved healing, whereas lower pressures are associated with poor prognosis or amputation [201]. While diabetes and hypertension frequently coexist, no standardized criteria link abnormal blood pressure directly to diabetes-related complications, emphasizing the need for multifunctional signal monitoring to control diabetes.

## Wearable Detection

### Wearable Chemical Biosensor

Wearable optical biosensors have gained prominence in personalized health care due to their ability to provide real-time, noninvasive monitoring of biological markers. They employ techniques such as fluorescence spectroscopy, surface plasmon resonance (SPR), and surface-enhanced Raman scattering (SERS) to detect analytes. These sensors rely on optical detection principles—including absorbance (colorimetric), emission (fluorescence or luminescence), and scattering (plasmonics)—to identify and quantify specific biomarkers in bodily fluids. For instance, glucose concentrations can be monitored via changes in color or fluorescence intensity, offering visual or measurable indicators of blood sugar levels.

While optical sensors offer high sensitivity to environmental changes and rely on light-based detection, electrochemical biosensors are more adaptable for real-world biological and environmental samples. Wearable electrochemical biosensors enable continuous, noninvasive monitoring of health parameters and are vital for real-time health assessment and personalized care. These sensors use specific biorecognition elements—such as enzymes, antibodies, or nanomaterials—on conductive surfaces to selectively interact with target biomarkers, producing measurable electrical signals. The signal originates from the movement of electrons or ions, generating outputs like current or potential changes.

Over the past two decades, wearable electrochemical biosensors have gained traction for their affordability, high sensitivity and selectivity, portability, and user-friendliness. They can perform reliably in complex environments and are excellent for early disease detection. Many are also reusable and easy to apply or remove.

#### Bioreceptors

Bioaffinity sensors typically comprise a bioreceptor layer for specific molecule recognition and a signal transducer, which transforms the interaction event between a target and a receptor into a measurable signal. Bioreceptors used in wearable sweat biosensors construction include antibodies, nucleic acids, enzymes, and biomimetic materials such as molecularly imprinted polymers (MIPs).

The choice of biorecognition element must align with the intended application to ensure sensor performance [67, 191]. Whether naturally derived or synthetically engineered, these elements mediate the molecular interactions that link biomarker presence to signal generation [67]. Integrating them into flexible wearable platforms involves overcoming challenges such as secure immobilization on flexible electrodes, maintaining structural integrity during use, automating sweat sampling, and enabling bioreceptor reuse without compromising function [66, 202].

##### Enzyme-Based Biosensors

Enzymes play a crucial role in wearable biosensors, offering high sensitivity for detecting small molecules like metabolites. Redox enzymes, such as glucose oxidase, are particularly valuable due to their catalytic activity, which enables signal amplification [202]. Most enzyme-based biosensors function by coupling catalytic reactions with redox processes, detecting either direct or mediator-assisted electron transfer to an electrode can be used to create specific substrate-to-product pathways for enhanced specificity [203]. Their catalytic efficiency makes them ideal for continuous monitoring, provided that product inhibition is well managed.

However, enzyme selection must be precise to avoid broad substrate specificity, which may cause cross-reactivity and ambiguous readings in complex biofluids. Other critical considerations include enzyme stability, ease of immobilization, and the risk of self-inactivation from redox by-products. Immobilizing enzymes effectively can also be chemically challenging [204, 205].

**Fig. 6:**
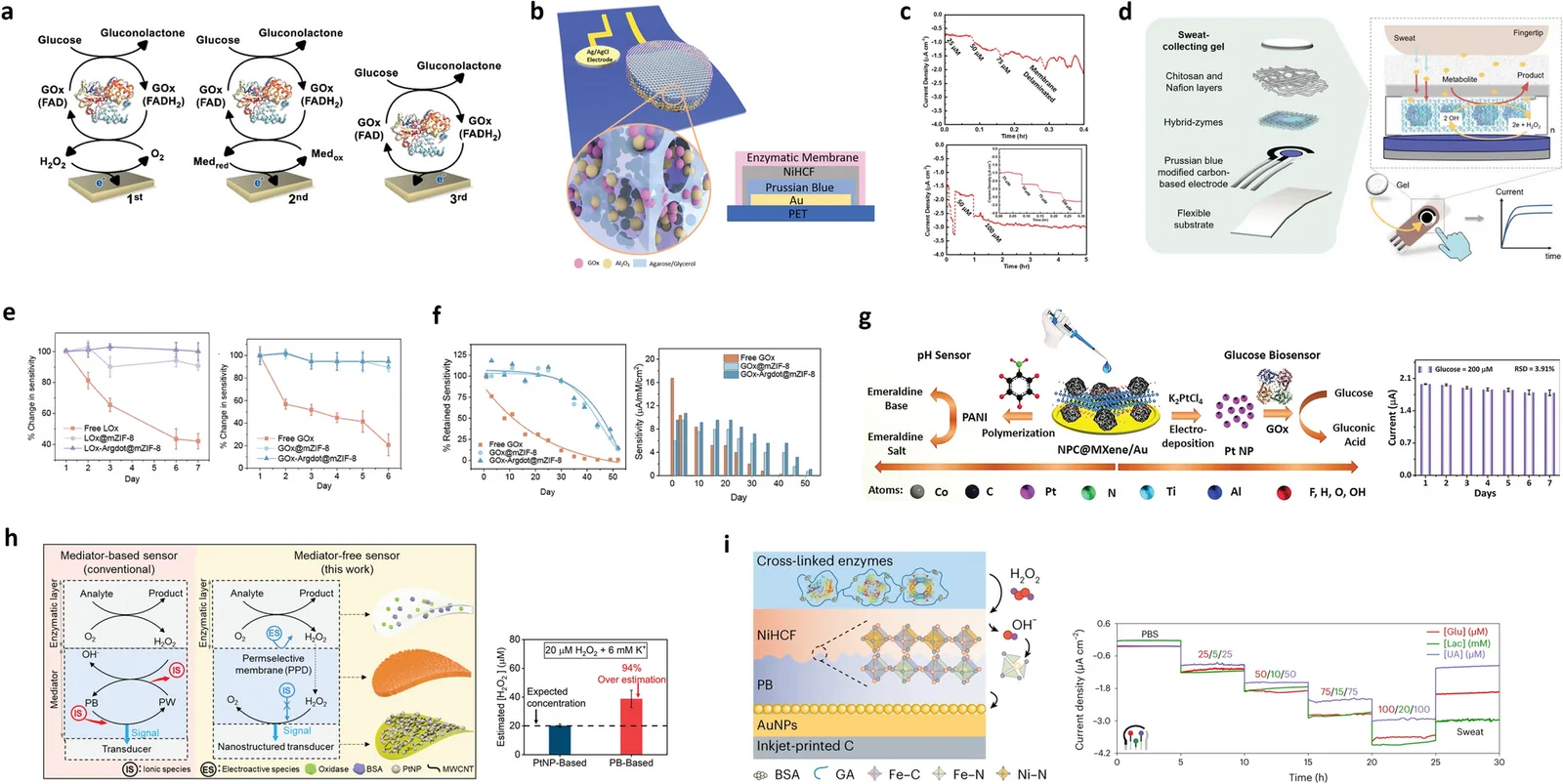
Enzymatic sensors. **a** Generations of electrochemical glucose biosensors. Reproduced with permission from Ref. [232]. Copyright 2024 American Chemical Society. **b** Schematic illustration of the nanotextured glucose sensors. **c** Working electrode and the glucose sensors with membrane delamination (left) and the nanotextured glucose sensors without membrane delamination (inset: amperometric response in the first 20 min) (right). Reproduced with permission from Ref. [212]. Copyright 2019 Wiley. **d** GOx-based sensor components and touch-based fingertip sweat glucose test setup. **e** Change in lactate and glucose sensitivity over one week. **f** Changes in glucose sensitivity over 50-day storage. Reproduced with permission from Ref. [213]. Copyright 2024 Wiley. **g** Working electrode functionalization process involves glucose and pH sensors. Long-term stability of the glucose biosensor. Reproduced with permission from Ref. [228]. Copyright 2022 Wiley. **h** Reaction schematic of a PB-based electroenzymatic sensor vs the mediator-free electroenzymatic sensor. Estimation of the H_2_O_2_ concentration level by PB-based and PtNP-based sensing interfaces in PBS. Reproduced with permission from Ref. [206]. Copyright 2020 Wiley. **i** Mechanism of the enzymatic metabolite sensors, and long-term stability of the enzymatic glucose, lactate and UA sensors in PBS and sweat samples. Reproduced with permission from Ref. [229]. Copyright 2024 Springer Nature

In sweat-based electrochemical sensing, enzymes immobilized on electrode surfaces drive electrocatalysis. For diabetes monitoring, glucose oxidase (GOx) and lactate oxidase (LOx) are commonly employed. Over time, enzyme-based sensors have evolved through three distinct generations (Fig. 6a), reflecting advancements in sensitivity, specificity, and electron transfer mechanisms.

The first-generation enzyme-based biosensors detect by-products of enzyme–substrate reactions. In glucose sensing, for example, hydrogen peroxide produced by GOx or glucose dehydrogenase (GDH) is measured. However, their reliance on oxygen as the electron acceptor caused signal variability under fluctuating oxygen levels [206]. To address this, researchers developed oxygen-rich carbon paste electrodes using fluorocarbon-based pasting liquids with high oxygen solubility [207], modified GOx to reduce oxygen dependency through amino acid engineering [208], and designed membranes with bidirectional oxygen diffusion to enhance performance [209].

Second-generation sensors replaced oxygen with synthetic redox mediators to shuttle electrons from enzyme to electrode, reducing operating potential and eliminating oxygen dependence. Common mediators include ferrocene, hydroquinone, tetrathiafulvalene, methylene blue, Prussian blue, and osmium-based redox hydrogels [210, 211]. However, the second-generation sensors faced stability issues and produced inconsistent readings over time. To address these challenges, researchers suggested using a porous membrane for more effective enzyme immobilization [212]. Additionally, strategies such as co-encapsulation with arginine-derived carbon dots (Argdots) [213] and the use of protective layers including chitosan and Nafion [214] have been employed to improve sensor stability and performance.

Third-generation sensors achieved direct electron transfer between enzymes and electrodes, removing the need for mediators. This was enabled by nanomaterials, carbon nanotubes (CNTs), and metal–organic frameworks (MOFs) [215, 216]. For instance, a flexible tattoo-based electrochemical biosensor using immobilized lactate oxidase was developed for real-time, noninvasive lactate monitoring during exercise [217]. It showed good selectivity and linearity up to 20 mM, while withstanding mechanical stress—key for wearable applications. However, direct electron transfer remains challenging due to the insulating protein shell of enzymes like GOx, which impedes direct electron tunneling from the redox center (e.g., FAD) to the electrode [210].

Sweat typically has a pH between 4.5 and 7.5 and a temperature range of 33.5–36.9 °C [218, 219]. These variations pose challenges for enzyme-based sensors, which are highly sensitive to pH and temperature fluctuations that can affect enzyme activity and measurement accuracy [220]. One study incorporated skin temperature measurement into a multiplex sensor (glucose, lactate, sodium, potassium) to calibrate and compensate for temperature-induced changes in enzyme activity, ensuring accurate readings despite temperature variations [221]. Other research addressed similar issues by integrating pH and temperature sensors for real-time calibration of glucose measurements under changing conditions [222].

Enzyme immobilization on the electrode surface is crucial for stable sensor performance. Chitosan, a biocompatible material with nitrogen and oxygen functional groups suitable for covalent enzyme attachment, is frequently employed as a transducer surface modifier [223]. Many enzyme-based electrodes for sweat sensing use chitosan to enhance immobilization [221, 224, 225], often in combination with other compounds (e.g., genipin) for increased stability. A lactate sensor featuring a chitosan–genipin membrane immobilizing LOx and a thionine mediator demonstrated stable enzyme activity and consistent signal output over a 1–50 mM lactate range [226].

Biosensors can also integrate hydrogels like agarose or polyvinyl alcohol (PVA) cryogels to maintain a stable pH and prevent enzyme denaturation [227]. In addition, introducing electron mediators such as Prussian blue (PB) facilitates efficient electron transfer and preserves enzyme functionality. Nevertheless, enzymatic biosensors face limitations due to the inherently poor stability of enzymes [2, 212]. Figure 6b, c illustrates a porous membrane anchored to nanotextured electrode contacts, effectively preventing enzyme escape while providing sufficient surface area for molecular diffusion, sustaining catalytic activities, and ensuring reliable, measurable signals during noninvasive monitoring [212]. The resulting nanoporous membranes and nanotextured electrodes exhibit enhanced sensing stability, mechanical robustness, and long-term amperometric performance.

Further innovations include co-encapsulating enzymes and arginine-derived carbon dots (Argdots) in a mesoporous Zeolitic Imidazolate Framework-8 (mZIF-8) matrix (Fig. 6d–f) [213]. This GOx-Argdot@mZIF-8 nanocomposite delivers 40% higher electrochemical sensitivity than GOx@mZIF-8 alone. Argdots enlarge mZIF-8 pores, reducing diffusion barriers and improving analyte access to enzyme active sites. Multiple protective layers, including chitosan and Nafion, also help prevent nanocomposite leaching.

Another approach involves immobilizing GOx on a nanoporous carbon and MXene heterostructure, paired with platinum nanoparticles for sweat glucose detection (Fig. 6g) [228]. This stable matrix preserves enzyme activity by shielding it from harsh conditions. Integrated pH and temperature sensors ensure accurate glucose measurements despite environmental changes, underscoring the importance of sensor design and protective strategies in enabling reliable, continuous monitoring of sweat biomarkers.

Conventional mediator-based sensors that rely on Prussian blue (PB) often exhibit limited stability due to PB degradation under varying pH conditions, undermining long-term reliability. Emaminejad’s group addressed this issue with a mediator-free sensing interface (Fig. 6h) [206], featuring a platinum nanoparticle (PtNP)/multiwall carbon nanotube (MWCNT) layer and a poly-m-phenylenediamine permselective membrane. Unlike PB, the Pt-based electrode is inert to ionic species, while the poly-m-phenylenediamine layer selectively permits small, neutral molecules (e.g., hydrogen peroxide) to reach the electrocatalytic surface. This design achieved minimal signal drift (< 6.5% over 20 h) and excellent long-term stability. In another strategy, researchers stabilized PB by depositing nickel hexacyanoferrate onto it, forming a solid solution composite that bolsters PB’s interface stability [229]. A resulting device monitored three vital signs—pulse waveform, galvanic skin response, and skin temperature—as well as six sweat biomarkers (glucose, lactate, uric acid, sodium, potassium, and ammonium), demonstrating stable glucose and lactate sensing over 100 h with minimal signal decay (< 0.07% per hour) (Fig. 6i).

The incorporation of nanomaterials with catalytic properties further enhances enzymatic sensor sensitivity [230]. For instance, noble metal electrodes (gold or platinum nanoparticle-coated) boost electrochemical activity and overall sensitivity [231]. Additionally, poly-m-phenylenediamine membranes serve as permselective layers, blocking interfering electroactive species so that only glucose and lactate reach the electrode.

Despite their widespread usage, enzymatic sensors face several significant challenges. The stability of enzymes and their sensitivity to changes in pH, ionic strength, and temperature are critical issues. Additionally, enzymatic self-inactivation and interference from other chemicals in the environment further complicate their use. While recent advancements, such as incorporating polymers and porous structures, have been made to address these issues, current solutions remain insufficient. Furthermore, effective enzyme immobilization and enhancing the electron transfer between the enzyme and the electrode surface are crucial areas that require further development.

##### Antibody-Based Biosensors (Immunosensors)

Antibodies are recognized as the principal bioreceptors employed in bioaffinity sensors, valued for their superior affinity and specificity to targets, versatility, and commercial availability [233]. Produced by the immune system, these proteins bind specifically to antigens. Monoclonal antibodies (from a single cell line) and polyclonal antibodies (from multiple cell lines) are commonly used. When the target antigen is present in a sample, it binds to the immobilized antibody, causing a change in the transducer’s physical or chemical properties. Antibody-based sensors can be labeled or label-free. Label-free immunosensors detect antigen–antibody interactions directly, converting their binding event into electrical or optical signals without requiring tracers [233].

**Fig. 7:**
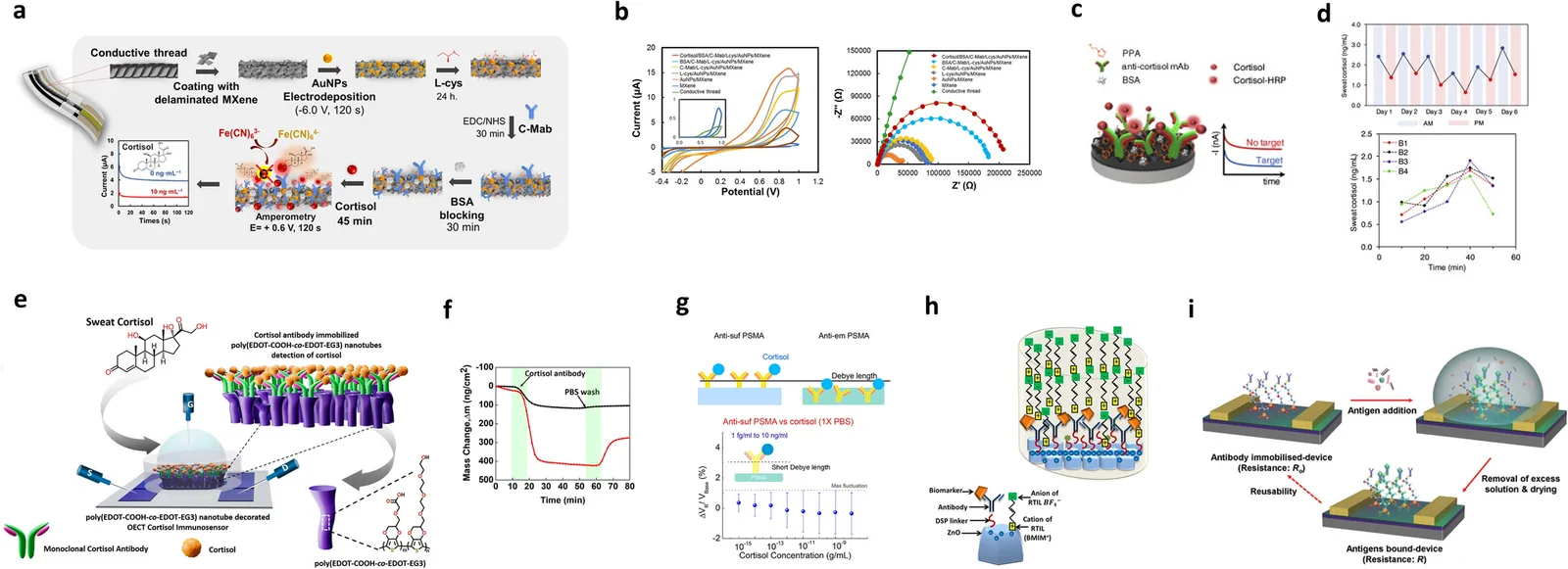
Antibody-based biosensors. **a** Illustration of the fabrication of the working electrode of the thread-based electrochemical cortisol immunosensor. **b** CV and EIS of the immunosensor. Reproduced with permission from Ref. [238]. Copyright 2022 Elsevier. **c** Construction and sensing strategy of the affinity-based electrochemical cortisol sensor. **d** Circadian rhythm of sweat cortisol for a healthy subject in 6 days, and cortisol monitoring from three physically untrained subjects (B1–B3) and one trained subject (B4) in a constant-load cycling exercise. Reproduced from Ref. [101] under CC BY 4.0. Copyright 2020 Torrente-Rodríguez et al. **e** Illustration of the detection of cortisol. **f** Mass change of the cortisol sensor, poly(EDOT-COOH-*co*-EDOT-EG3)-coated OECT channel layers (black line) and poly(EDOT-COOH-*co*-EDOT-EG3) nanotube-coated OECT channel layers (red line). Reproduced with permission from Ref. [242]. Copyright 2022 American Chemical Society. **g** Schematic of antibody-embedded geometric in the PSMA polymer matrix, and Δ*V*_R_ of antisuf PSMA in different concentrations of cortisol. Reproduced with permission from Ref. [243]. Copyright 2018 American Chemical Society. **h** A schematic of the sweat-based wearable diagnostic biosensor. Reproduced from Ref. [246] under CC BY 4.0. Copyright 2017 Munje et al. **i** Depiction of the summary of the biosensing technology. Reproduced with permission from Ref. [248]. Copyright 2021 Wiley

Antibodies play a crucial role in measuring hormones in sweat including cortisol. Monoclonal antibodies specific to cortisol, displaying high affinity and specificity, are commonly employed [234–236]. Electrochemical impedance spectroscopy (EIS) and voltammetry methods often characterize performance [235, 236]. One sensor used cyclic voltammetry (CV) and differential pulse voltammetry (DPV) to detect cortisol at concentrations as low as 0.005 fg mL^−1^ [237]. This heightened sensitivity was attributed to the large surface area provided by ellipsoidal Fe_2_O_3_ nanostructures on carbon yarn. In another study, a conductive thread electrode was modified with L-cysteine, gold nanoparticles, and MXene before antibody immobilization (Fig. 7a, b) [238]. This approach boosted surface area and electron transfer, yielding a wide linear range (5–180 ng mL^−1^) and low detection limit (0.54 ng mL^−1^). The sensor successfully measured cortisol within the normal physiological range.

Achieving stable, effective antibody immobilization while preserving bioactivity is critical for accurate cortisol sensing. Working electrodes are frequently functionalized for covalent antibody attachment, often using 1-ethyl-3-(3-dimethylamino)propyl carbodiimide/N-hydroxysuccinimide (EDC/NHS) coupling. For example, MOFs were used to orient and immobilize antibodies so that their antigen-binding regions remained exposed [239]. Alternatively, thiol-based functionalization ensures strong covalent bonding to gold electrodes [240, 241].

One noteworthy advance involved a pyrrole-derivative graft modified on a graphene surface prior to monoclonal anti-cortisol antibody immobilization (Fig. 7c, d) [101]. After polymerizing 1H-pyrrole propionic acid on a laser-induced graphene electrode, EDC/NHS activation enabled covalent bonding of the antibodies. A competitive immunosensing approach, using horseradish peroxidase (HRP)-labeled cortisol, achieved a detection limit of 0.08 ng mL^−1^.

Transistor platforms offer high sensitivity and specificity in cortisol detection. One study developed an organic electrochemical transistor (OECT) functionalized with poly(3,4-ethylenedioxythiophene) (PEDOT) derivatives, forming a bilayer channel (Fig. 7e, f) [242]. The nanostructured upper layer provided extra surface area for antibody binding, enabling detection from 1 fg mL^−1^ to 1 μg mL^−1^ with a detection limit of 0.0088 fg mL^−1^. Another sensor employed a field-effect transistor (FET) design with a poly(styrene-co-methacrylic acid matrix to embed antibodies, effectively addressing Debye length limitations in high-ionic-strength solutions (Fig. 7g) [243]. This system achieved a linear range of 10 fg mL^−1^ to 10 ng mL^−1^ with a detection limit of 1 pg mL^−1^.

Sweat immunosensors have also been employed for monitoring cytokines [244, 245]. For instance, one design used non-faradaic EIS to detect Interleukin-1 beta (IL-1β) and C-reactive protein with detection limits of 0.2 pg mL^−1^ [244]. Another employed ionic liquids (e.g., 1-butyl-3-methylimidazolium) to stabilize anti-IL-6 antibodies on ZnO thin films (Fig. 7h) [246]. Additional strategies include a thiol cross-linker for antibody immobilization [247] and (3-glycidyloxypropyl)trimethoxysilane functionalization on ZnO thin films, forming stable, ordered layers for antibody attachment (Fig. 7i) [248].

Despite challenges such as production costs, stability issues, and potential batch-to-batch variation, antibodies remain integral to current biosensing technologies due to their exceptional sensitivity and selectivity for biomarkers [249]. Ensuring the stability and bioactivity of immobilized antibodies over time is a significant challenge, as is maintaining sensor performance under mechanical stress and enabling continuous, real-time monitoring. Future research may focus on developing nanobodies, which are more compact and heat-stable, to address issues related to the storage and implementation of current immunosensors [249]. Additionally, the development of nonanimal-derived antibodies could enhance versatility and reproducibility while reducing ethical concerns [250].

##### Nucleic Acid-Based Sensors

Aptamers are single-stranded nucleotides (DNA, RNA, or xenobiotic alternatives) selected in vitro for their high-affinity binding to diverse targets, including ions, small molecules, proteins, and cells [251]. They offer several advantages over antibodies—such as cost-effectiveness, consistent quality, and ease of chemical modification—while demonstrating minimal immunogenicity and excellent stability [252, 253]. Their synthetic flexibility enables broad applicability across various sensing platforms [254].

Aptamers are typically generated through the Systematic Evolution of Ligands by Exponential Enrichment (SELEX), an iterative process of incubation, binding, partitioning, and amplification [255]. Effective surface immobilization is essential for stable performance. For instance, a 5′-thiol-terminated ssDNA aptamer targeting cortisol formed a robust self-assembled monolayer on ZnO and silver electrodes [256]. This chemisorption ensured reliable aptamer attachment throughout sensor operation.

**Fig. 8:**
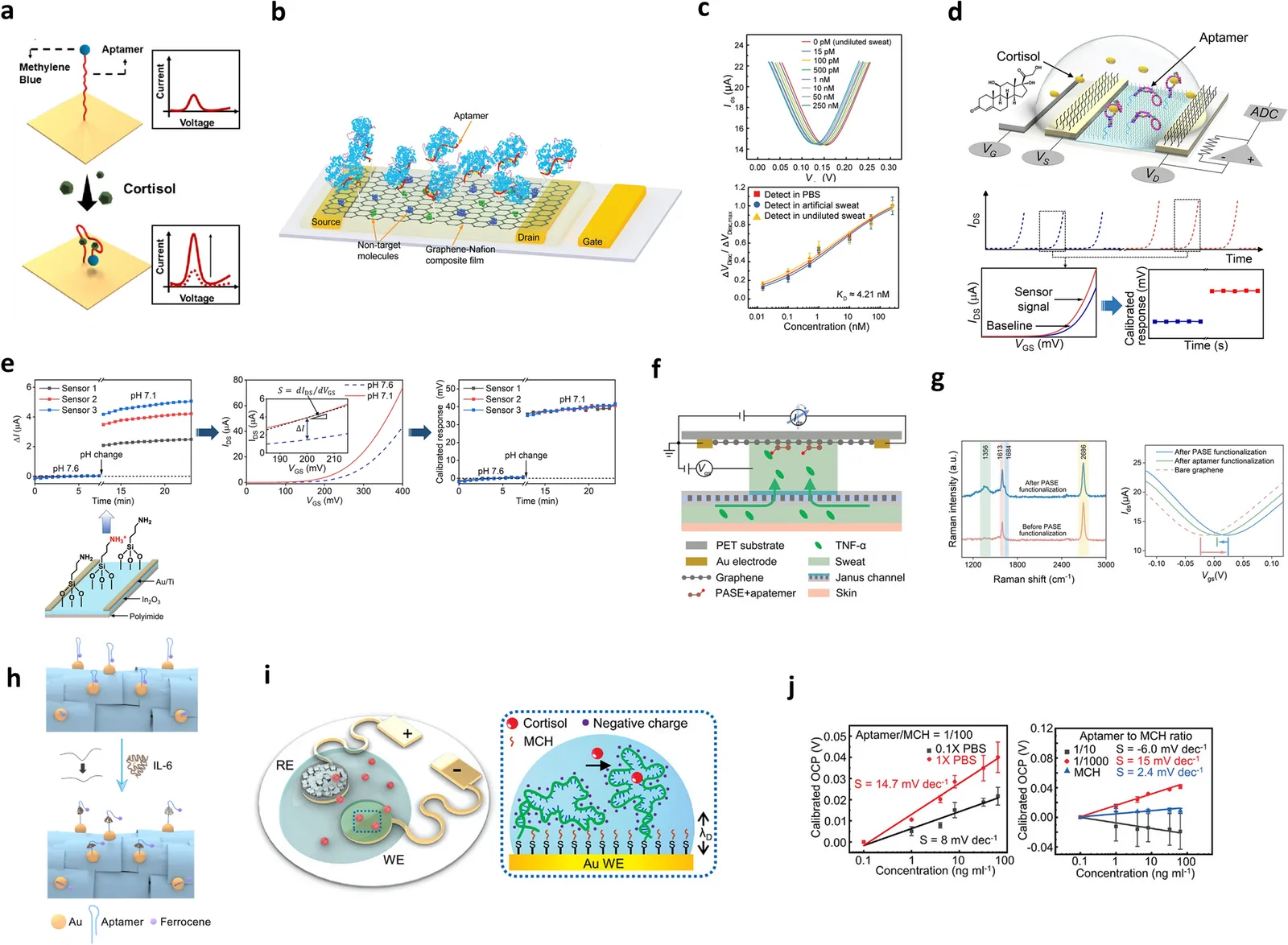
Sweat aptasensor. **a** Schematic of the functionalized corrugated sensor surface. Reproduced with permission from Ref. [257]. Copyright 2023 American Chemical Society. **b** Schematic of the aptameric GNFET biosensor. **c** Transfer characteristic curves and normalized Δ*V*_Dirac_/Δ*V*_Dirac, max_ as a function of different IFN-γ concentrations. Reproduced with permission from Ref. [260]. Copyright 2021 Wiley. **d** Schematic illustration of cortisol sensing by the aptamer-FET sensor. *V*_G_, gate voltage; *V*_D_, drain voltage;* V*_S_, source voltage; ADC, analogue–digital converter. **e** Bottom: channel surface charge perturbation mechanism, where primary amine groups of APTES self-assembled on In_2_O_3_ and were protonated with decrease in pH. Top-left: Real-time *I*_DS_ changes (Δ*I*) of FET-based pH sensors upon decreasing the solution pH. Middle: Calculation of FET calibrated responses by dividing absolute sensor responses (Δ*I*) by the slope (*S* = d*I*_DS_/d*V*_GS_) to reduce device-to-device variation. Right: calibrated FET pH responses. Reproduced from Ref. [100] under CC BY 4.0. Copyright 2022 Wang et al. **f** A principle diagram of the sensor for TNF-α detection. **g** Raman spectra of graphene before and after PASE modification and changes in the current–voltage curve. Reproduced with permission from Ref. [261]. Copyright 2024 Wiley. **h** Schematic of the sensing mechanism for IL-6 detection based on conformal change of the aptamer on the CNT/graphene composite fiber. Reproduced with permission from Ref. [262]. Copyright 2023 Elsevier. **i** Schematic illustration of the sensing mechanism of the anti-cortisol aptamer functionalized electrodes. The aptamers undergo a conformational change upon binding with cortisol. **j** Calibration plots of working electrodes in solutions with varying cortisol concentrations (left). Calibration plots of working electrodes with different aptamer/MCH ratios (right). Reproduced from Ref. [263] under CC BY 4.0. Copyright 2022 Dong et al.

Compared to protein-based receptors, aptamers’ smaller binding footprint helps reduce nonspecific interactions. In a cortisol sensor, incorporating methylene blue as a redox reporter allowed direct measurement of binding events with low background signals (Fig. 8a) [257]. A pseudoknot-assisted aptamer design further enhanced specificity by restricting the aptamer to discrete bound and unbound states, ensuring that the redox signal changed mainly in response to target binding [258].

Field-effect transistors (FETs) integrated with aptamers have been developed for sweat biomarker detection [100, 259]. One graphene–Nafion FET demonstrated high selectivity for interferon-gamma (IFN-γ) with femtomolar detection limits (Fig. 8b, c) [260]. Still, aptamer-FETs can experience variability due to pH shifts or inconsistencies among devices. To address these issues, a self-referencing method was implemented in a cortisol aptasensor [100]. Initially, transfer curves were recorded for each FET by sweeping the gate–source voltage (V_GS) at a constant drain–source voltage (V_DS). The sensor response to pH changes was then evaluated by measuring the absolute change in the source–drain current (I_DS). The slope of the I_DS–V_GS transfer curve at the chosen operating point (V_GS bias) served as an internal sensitivity measure. By dividing the sensor response (Δ*I*) by this slope (*S*), the responses could be normalized, achieving consistency across different FETs, significantly reducing variability, and improving the reliability of the readings (Fig. 8d, e).

Surface modification of an aptamer-functionalized graphene FET sensor is critical to its performance. One commonly used modifier, 1-pyrenebutanoic acid succinimidyl ester (PASE), interacts with graphene through *π*–*π* interactions, ensuring the stability and functionality of the graphene layer [259, 261]. In one cytokines sensor, PASE stabilized the graphene surface and allowed for aptamer attachment [261]. Subsequently, a shift in the Dirac point was observed, indicating n-type doping by PASE. This shift preceded the p-type doping induced by the aptamer, a key step for the sensor’s functionality (Fig. 8f, g). Furthermore, PASE enhanced sensor performance for cytokine detection in undiluted sweat at concentrations ranging from 0.5 to 500 pM, achieving a detection limit of 0.31 pM.

Aptamers can also be engineered with redox-active reporters to generate electrochemical signals. In one example, a ferrocene-modified aptamer was covalently immobilized onto a fiber substrate via Au–S binding [262]. This modification was essential because the conformational change of the ferrocene-modified aptamer upon binding to interleukin-6 (IL-6) resulted in a decrease in the ferrocene redox signal (Fig. 8h). The study proposed using aptamer-functionalized carbon nanotube/graphene composite fibers to create a wearable electrochemical fabric for real-time IL-6 monitoring.

Electrostatic repulsion among negatively charged oligonucleotides can affect the aptasensor’s sensitivity by altering aptamer conformation at the sensor–solution interface [263, 264]. A recent study addressed this by developing a fully passive sensing interface, functionalizing gold surfaces with single-stranded DNA aptamers that bind cortisol [263]. The conformational changes triggered by cortisol binding shifted the surface potential of the working electrode (Fig. 8i). Systematic analyses revealed that the ratio of aptamer to 6-mercapto-1-hexanol (MCH) greatly influenced sensor sensitivity (Fig. 8j). An aptamer-to-MCH ratio of 1/100 proved optimal, balancing sensitivity with reduced electrostatic repulsion. For aptamer immobilization, the anti-cortisol DNA aptamer was reduced with dithiothreitol, followed by drop-casting onto the gold electrode to form stable Au–S bonds. Another study corroborated that a 1/100 ratio was effective in minimizing the effects of electrostatic repulsion [264].

Despite the range of aptamer-based sensing platforms, widespread clinical application remains limited. Challenges include improving the signal-to-noise ratio, ensuring high-confidence signal detection, and overcoming the cost and instability of DNA/RNA aptamers—many of which degrade in sweat due to enzymatic activity. Strategies to address these include introducing biocompatible functional groups or using more stable xeno nucleic acids (XNAs) to reduce costs and enhance resilience [265]. In addition, there is a need to improve the sensitivity of aptasensors, particularly for detecting low concentrations of target molecules in complex samples. This can be achieved by integrating aptamers with nanomaterials and employing signal amplification strategies. More research and development efforts are needed to overcome the challenges related to their susceptibility to nucleases and the cost of their mass production.

##### Molecularly Imprinted Polymers-Based Sensors

Molecularly imprinted polymers (MIPs) are synthetic bioreceptors that mimic biological recognition systems, designed by polymerizing functional monomers around specific template molecules. After polymerization, the template is removed, leaving behind selective binding sites complementary to the original molecule, enabling targeted analyte recognition [266]. MIPs offer significant advantages over biological receptors such as antibodies or enzymes, including greater durability, stability under harsh conditions, and cost-effectiveness. However, a notable limitation of MIPs is their inherent lack of intrinsic signaling or catalytic properties, requiring integration with external signal-transduction mechanisms to translate the binding events into measurable responses [60, 267].

**Fig. 9:**
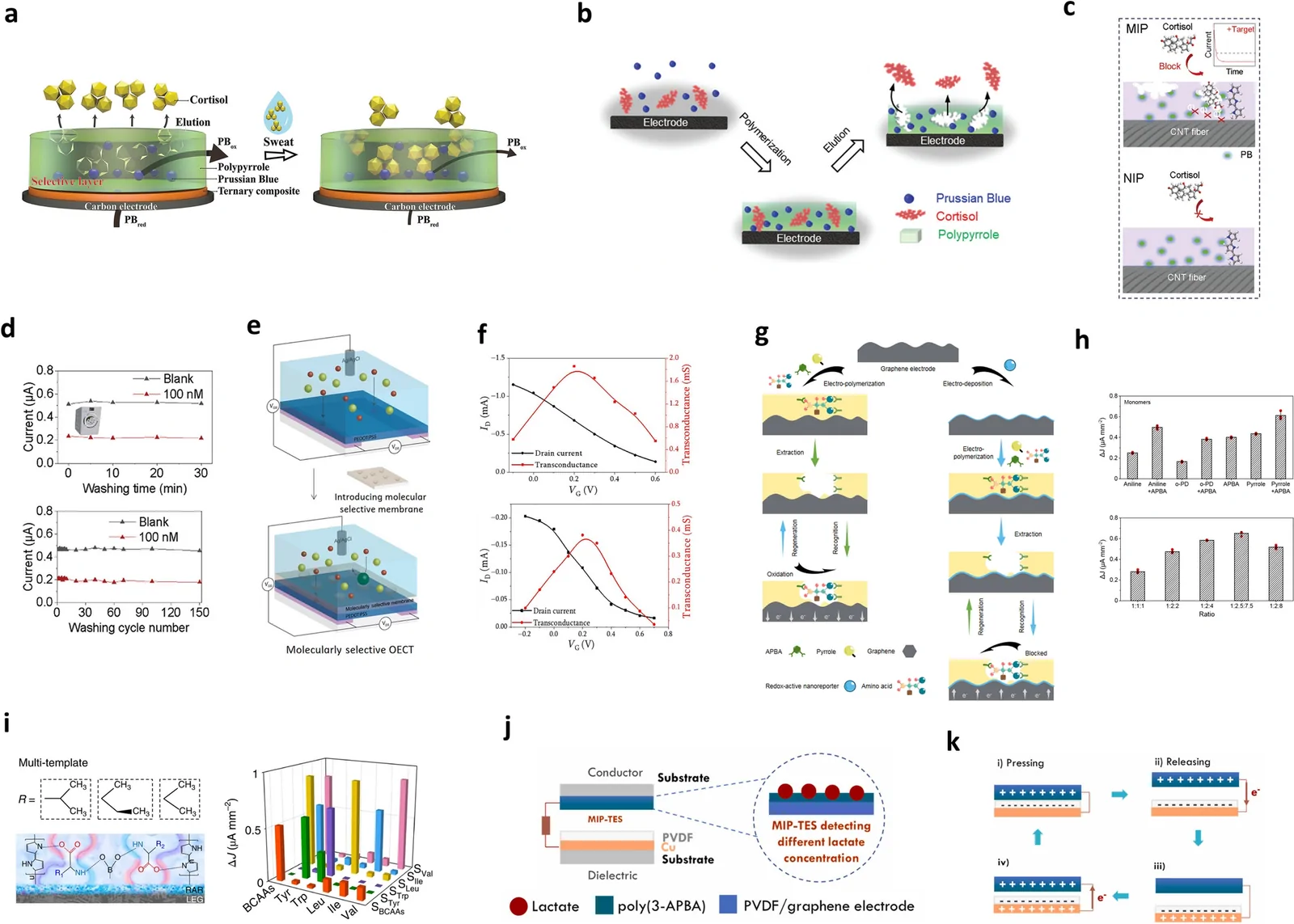
MIP-based sensors. **a** Sensing mechanism of the cortisol sensor. Reproduced with permission from Ref. [269]. Copyright 2023 Wiley. **b** Co-electrodeposition of PB, cortisol, and pyrrole onto the printed carbon electrode. Reproduced with permission from Ref. [278]. Copyright 2021 Wiley. **c** Schematic illustration of the detection of cortisol. **d** Current response of the sensor before and after washing. Reproduced with permission from Ref. [283]. Copyright 2024 Wiley. **e** Schematic illustration of the integration of MSM to OECT device consisting of PEDOT:PSS channel coated on an indium tin oxide glass substrate with the cortisol-selective membrane. **f** Device characteristics demonstrated by transfer curves before and after MSM integration. Reproduced from Ref. [285] under CC BY 4.0. Copyright 2018 Parlak et al. **g** Schematic of the preparation and detection mechanism of the sensors. An electroactive amino acid sensor with a direct detection mechanism (left) and an amino acid sensor with an indirect detection mechanism (right). **h** Current density of the peak height (ΔJ) of the tryptophan (Trp) MIP sensors based on different monomer/target combinations (top) and different target/cross-linker/monomer ratios (bottom). **i** Schematic of multi-MIP amino acid sensors (left) and selectivity of different amino acid sensors against other amino acids (right). Reproduced with permission from Ref. [76]. Copyright 2022 Springer Nature. **j** Schematic illustration of the lactate sensor. **k** Working principle for the electrical generation of the electrical signal. Reproduced with permission from Ref. [286]. Copyright 2022 Elsevier

Conducting polymers enhance electrochemical MIP sensors due to their intrinsic conductivity, facilitating efficient electron transfer. Notable examples include lactate sensors created by electropolymerizing 3-aminophenylboronic acid (3-APBA) onto silver nanowire electrodes [268]. and cortisol sensors employing polypyrrole (PPy) integrated with MXenes, carbon nanotubes (CNTs), or silver nanoparticles, significantly enhancing sensor sensitivity and performance (Fig. 9a) [269].

Nonconductive polymers, such as methacrylic acid, also effectively enhance sensor performance when combined with conductive nanomaterials [270–272]. For example, copolymerizing methacrylic acid with methyl methacrylate (MMA) onto gold nanoparticle-modified graphene oxide significantly improved conductivity and sensitivity, achieving exceptionally low detection limits for biomarker detection [270]. Additionally, glucose-templated MIPs incorporating gold nanoparticles demonstrated excellent stability and sensitivity, achieving a detection limit of 1.25 nM [273].

Various MIP fabrication methods exist, including electropolymerization, photopolymerization, bulk polymerization, and precipitation polymerization, each method offering distinct advantages [274]. Recent advancements, such as two-photon polymerization stereolithography and bioprinting, allow precise spatial control over MIP structures, yielding enhanced surface areas and increased binding capacities, further improving sensor performance [275–277].

Prussian blue (PB) possesses excellent redox characteristics due to its reversible Fe^2^⁺/Fe^3^⁺ redox couple, facilitating efficient electron transfer processes. PB can be integrated directly into MIP matrices during polymerization, serving as an embedded redox probe. For instance, PB was employed in a label-free amperometric cortisol detection sensor (Fig. 9b) [278]. Cortisol binding to imprinted cavities impeded electron transfer pathways, resulting in decreased PB oxidation current, directly correlating with cortisol concentrations, eliminating the need for additional labeling.

In other studies, PB layers were electrodeposited onto electrode surfaces before MIP application [279, 280]. Specifically, PB nanoparticles were electrodeposited on screen-printed carbon electrodes prior to electropolymerizing pyrrole and 3-aminophenylboronic acid (3-APBA) as MIP layers [280]. Optimizing PB layer thickness ensured robust signal intensity and lactate response, while adequately covering PB nanoparticles with the MIP layer.

Redox-active nanomaterials beyond PB have also enhanced sensor performance. For example, copper phthalocyanine tetrasulfonate (CuPcTS) doped into polypyrrole demonstrated electrocatalytic activity, facilitating cortisol reduction by transferring hydride ions from the copper center to cortisol's ketone group [281]. Similarly, vinylferrocene was copolymerized with pyrrole-*co*-(dimethylamino)pyrrole for detecting cortisol and dehydroepiandrosterone (DHEA), leveraging ferrocene’s reversible oxidation–reduction characteristics to measure analyte concentrations through current changes [282].

Wearable biosensors’ washability and breathability significantly enhance their practical usability. Unlike biological receptors, MIPs maintain activity under harsh conditions. A fabric-based cortisol sensor demonstrated superior washability and breathability, incorporating CNT fiber electrodes functionalized with MIPs and redox-active nanoreporters (Fig. 9c) [283]. It exhibited high air permeability (47.4 mm s^−1^) and moisture permeability (10,091.9 g m^−2^ day^−1^), outperforming conventional PDMS films. The sensor maintained stable cortisol detection after washing with ethanol for up to 150 cycles (Fig. 9d).

Functionalization of electrodes further enhances MIP adhesion and sensor stability. For interleukin-6 (IL-6) detection, Au/screen-printed carbon electrodes were modified with 3-aminopropyltriethoxysilane (APTES), introducing amino groups reactive with cross-linking agents [284]. Subsequent glutaraldehyde treatment introduced aldehyde functionalities, enabling covalent bonding with biomolecules. This cross-linked polymer matrix significantly enhanced electrode durability, preventing template leaching and improving sensor reliability.

The developed transistor-based MIP biosensor facilitates highly sensitive detection of biomarkers in biological fluids. A recent study introduced a molecularly selective membrane (MSM) composed of MIPs integrated into an organic electrochemical transistor (OECT) (Fig. 9e) [285]. This MSM was created by embedding MIPs into a PVC matrix applied over the PEDOT channel, which modulated ion transport to the channel. Without cortisol, ions moved freely, altering channel conductivity. Cortisol binding restricted ion transport, significantly changing the electrical output. Figure 9f illustrates sensor characteristics through transfer curves before and after MSM integration.

Density functional theory (DFT) analysis has been utilized to improve MIP sensor selectivity by evaluating monomer–target interactions computationally, thus guiding optimal monomer and cross-linker selection for MIP synthesis (Fig. 9g, h) [76]. This predictive capability allows for the creation of highly selective, regenerable MIP sensors suitable for continuous wearable monitoring. For non-electroactive targets such as branched-chain amino acids (BCAAs), sensors employed indirect detection strategies involving redox-active reporter layers, such as Prussian blue or 2-anthraquinone carboxylic acid, prior to MIP deposition (Fig. 9i). BCAA binding to the MIP obstructed electron transfer from these reporters, resulting in a measurable decrease in redox signals. These sensors demonstrated exceptional selectivity even in complex sweat environments at physiologically relevant concentrations.

Triboelectric nanogenerators (TENGs) have been integrated with MIP technology to create self-powered triboelectric sensors (MIP-TES). One notable example utilized MIP-based sensors for lactate detection by modifying flexible PVDF/graphene electrodes with lactate-imprinted 3-aminophenylboronic acid (3-APBA) (Fig. 9j) [286]. The incorporated TENG converted mechanical movements into electrical signals, visually indicated by LED illumination powered directly by the sensor (Fig. 9k). This MIP-TES exhibited high selectivity and stability, significantly outperforming non-imprinted polymer (NIP) controls.

MIP-based biosensors offer greater stability and mechanical robustness compared to enzymatic, aptameric, or antibody-based alternatives. However, MIPs stored in aqueous or biological environments can suffer from swelling, structural degradation, or biomolecule adsorption [287].​ Addressing these storage and regeneration challenges remains critical. Direct electrochemical detection methods using MIPs often encounter interference from nonspecific interactions, prompting the integration of redox probes or enzymes to enhance sensitivity and specificity, although this may compromise stability. Additionally, cross-sensitivity issues with structurally similar compounds necessitate complex sensor arrays or setups for accurate discrimination. Environmental conditions, such as solvent composition, pH, and temperature, also significantly impact sensor performance, emphasizing the need for rigorous control during measurements [288, 289]. Table 4 summarizes various biorecognition elements used in wearable biosensor technologies.

**Table 4:** Bioreceptors of wearable electrochemical biosensors

Bioreceptor	Detection mechanism	Recognized biomarkers	Chemical functionalization	Continuous measurement	Advantages	Disadvantages	Referencess
Enzyme	Enzymatic reaction	Glucose, Lactate	Poor	Good	High specificity, catalytic signal amplification, continuous monitoring	Low stability, difficult immobilization, loss of activity, limited target choices, low sensitivity	[204, 290, 291]
Antibody	Direct antibody-antigen binding	Cytokines, cortisol	Poor	Poor	High specificity and sensitivity, commercial availability, high binding affinity	Expensive, possible batch-to-batch variability, stability issues, hard to regenerate	[233, 249, 290]
Nucleic acid	Complementary base pairing	Cytokines, cortisol	Good	Good	High specificity, less likely to elicit immune response, good sensitivity	Stability issues in complex biological matrices, high production cost, hard to regenerate	[252, 290, 292]
Molecularly imprinted polymers (MIPs)	Specific size, shape, surface chemistry memory	Glucose, lactate, cortisol, cytokines, BCAAs	Good	Good	High stability, easy synthesis, low cost, mass-producible, good selectivity	Lower binding affinity compared to biological receptors	[76, 266, 274, 290]

### Physical/Physiological Biosensor

#### Sweat Rate Sensor

Human sweat loss rates vary from approximately 10–2000 g m^2^ h⁻^1^ [293]. Sweat rate sensors quantify sweat production over time, offering critical data on sweat flux, which helps calibrate biomarker concentrations in sweat. Metabolite concentrations in sweat can be influenced by sweat rate variations; higher sweat rates often dilute metabolites, resulting in lower concentrations [60]. Accurate measurement of sweat rates ensures precise and reliable biochemical data, essential for monitoring dehydration, electrolyte balance, and overall health during physical activity. Sweat rate sensors are also valuable in dermatological and cosmetic assessments, evaluating skin hydration and product efficacy.

**Fig. 10:**
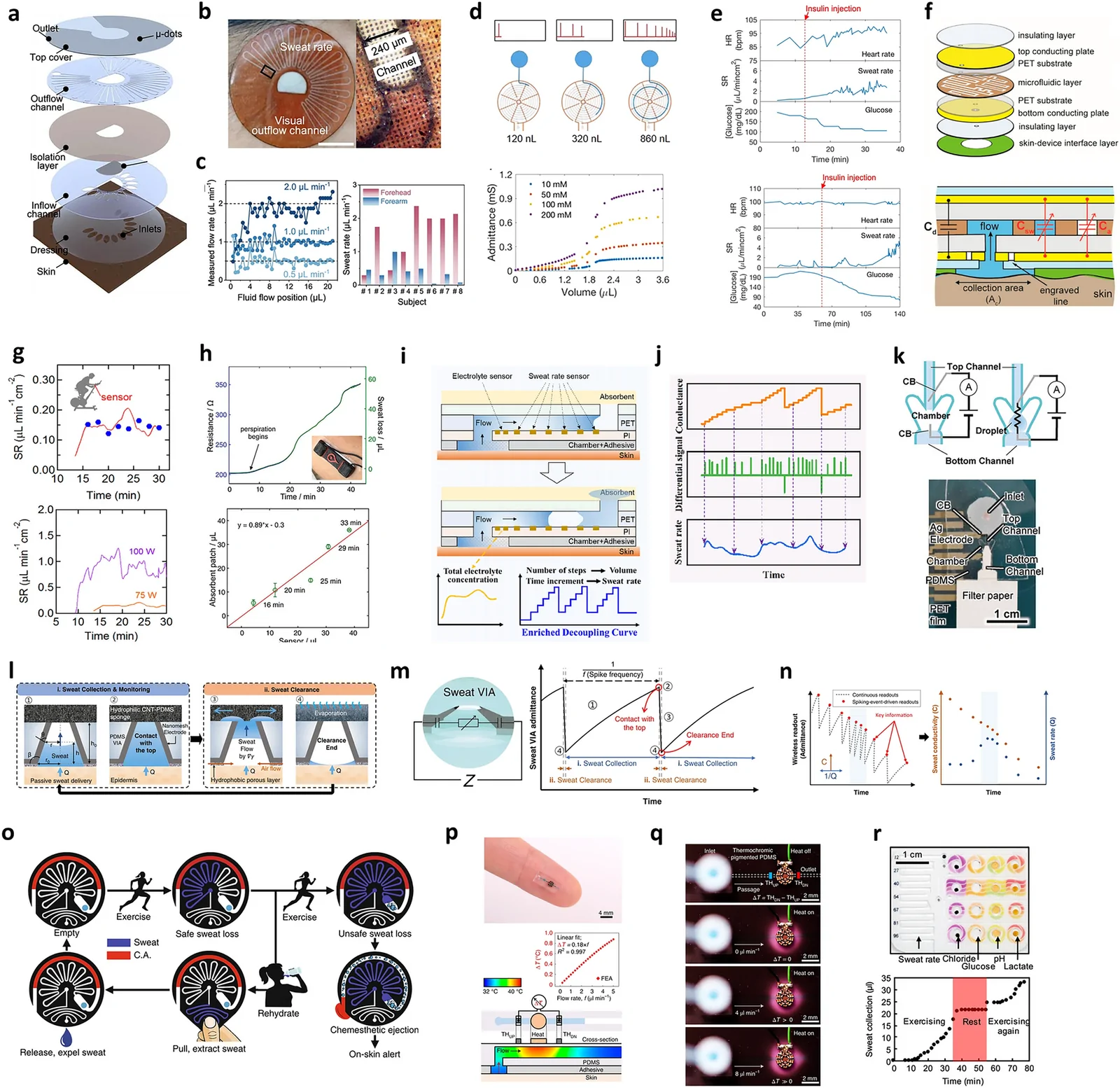
Sweat rate sensor. **a** Layer-by-layer view of the microfluidic device. **b** Photographs of the microfluidics during exercise and optical micrographs of sweat flowing in the microchannel. **c** Measured flow rates at different fluid-filling positions (left). Sweat rates were measured in eight healthy subjects during 10 to 20 min of exercise (right). Reproduced from Ref. [294] under CC BY 4.0. Copyright 2024 Zhong et al. **d** An impedimetric sweat rate sensing electrode and admittance responses to a solution containing NaCl. **e** Sweat secretion rate was measured along with heart rate and ISF glucose levels of a diabetic subject. Reproduced with permission from Ref. [296] under CC BY 4.0. Copyright 2021 Nyein et al. **f** Schematic illustration of the sweat rate sensor. The cross section illustration of the sensor and the equivalent electrical circuit elements (*C*_sw_: capacitance of the area of the microfluidic channel filled with sweat, *C*_a_: capacitance of the area of the microfluidic channel filled with air, *C*_d_: capacitance of the insulating area). **g** Sweat rate measured during exercise while the subject was spinning at 75 W intensity (red: sweat sensor, blue: macroduct) (left) and sweat rate response for a subject spinning at 75 and 100 W (right). Reproduced with permission from Ref. [300]. Copyright 2020 American Chemical Society. **h** Real-time monitoring of the perspiration dynamics during 45 min of high intensive cycling (left) and comparison of sweat loss values between the wearable sensor and regional absorbent-patch method (right). Reproduced with permission from Ref. [301] Copyright 2019 Wiley. **i** Schematic diagram of the principle of the sweat patch to decouple sweat information through the droplet separation strategy. **j** Diagram of decoupling the conductance “step” signal to extract sweat rate information. Reproduced with permission from Ref. [302]. Copyright 2024 Elsevier. **k** Schematic and the photo of the sweat rate sensor. Reproduced with permission from Ref. [303]. Copyright 2023 Wiley. **l** Schematic diagrams of the structures and operating principles of the sweat sensor. **m** Sensor’s equivalent circuit is an example of a spike admittance curve produced by the sweat sensor. **n** Illustration of spiking-event-driven data transmission. Reproduced from Ref. [304] under CC BY 4.0. Copyright 2022 Kim et al. **o** Schematic flow of a device operation. Reproduced from Ref. [305] under CC BY 4.0. Copyright 2019 Reeder et al. **p** Photograph of a thermal flow sensing module on a finger. Finite-element analysis of the temperature distribution through the passage filled with sweat flowing. **q** Images of a thermal actuator on a fluid passage illustrate the heat transfer and flow in the passage. Reproduced with permission from Ref. [306]. Copyright 2021 Springer Nature. **r** Epifluidic system with multiple colorimetric assays integrated with capillary bursting valves for analyzing sweat composition and rate (top). Cumulative local sweat loss versus time measured from the forearm during exercise and rest (bottom). Reproduced from Ref. [307] under CC BY 4.0. Copyright 2018 Choi et al.

Variability in individual sweat composition complicates interpreting sweat-based data and correlating it with blood analyte levels. To address this, a wearable multimodal biochip was developed, capable of simultaneously measuring phenylalanine (Phe), chloride concentrations, and sweat rate [294]. By normalizing Phe levels to sweat rates, the biochip reduced interindividual variability, enabling accurate correlation between sweat and blood biomarker levels. The biochip included a vertically integrated microfluidic module with pH buffering for stable biomarker detection (Fig. 10a–c). Sweat volume and rate were calculated in real-time via optimized microchannel filling and visual serpentine channels, aided by a computer vision algorithm that quantified sweat loss through color changes during exercise.

Another sensor system employed circular microfluidic channels fabricated through laser engraving, providing efficient and high temporal resolution sweat sampling by continuously delivering fresh sweat to sensors [295]. Sweat rate was determined by tracking sweat volume movement over time. This system also continuously monitored uric acid, tyrosine, skin temperature, and respiration rate in sweat.

Impedance-based sensors are prevalent to measure sweat rate in real-time [296–298]. For instance, an impedance-based sensor integrated interdigitated electrodes within microfluidic channels to detect changes in sweat flow by measuring admittance (inverse of impedance) as sweat contacted the electrodes [296]. This design enabled precise, real-time determination of sweat volume and flow rate, supporting continuous physiological monitoring (Fig. 10d). Then, hypoglycemia-induced sweat secretion was analyzed in a diabetic subject using a microfluidic patch worn on the finger, paired with a pulse oximeter (Fig. 10e). Measurements were conducted without disrupting routine insulin injections, demonstrating that significant reductions in glucose levels correlated strongly with increased sweat rates, whereas heart rate changes were less pronounced.

Capacitive sensors also enable continuous, real-time sweat rate monitoring by detecting capacitance changes as sweat fills microfluidic channels [299, 300]. A representative sensor employed two parallel conductive plates separated by plastic insulating layers surrounding a central microfluidic channel (Fig. 10f) [300]. As sweat replaced air in the channel, capacitance increased due to sweat’s higher permittivity. This capacitance change was directly proportional to sweat volume, enabling precise sweat rate measurements. The capacitive sensor demonstrated reliable performance during chemically and exercise-induced sweating (Fig. 10g).

Chemiresistors measure changes in electrical resistance correlating with water absorption. A sensor utilizing single-walled carbon nanotubes (SWCNTs) and sodium dodecylbenzenesulfonate as a surfactant detected linear increases in resistance as the paper substrate absorbed sweat [301]. Sweat absorption reduced hole density in the p-type SWCNTs, increasing resistance. Additionally, cellulose fiber swelling further increased resistance by enlarging gaps between nanotubes. This calibration-free approach enhanced operational simplicity and robustness. Device response during exercise and sweat loss validation are shown in Fig. 10h.

Conductance-based sensors (galvanic skin response or GSR sensors) measure changes in skin conductance to determine sweat rate, as sweat electrolytes enhance skin conductivity. A device utilized conductance "step" signals within a microchannel containing interdigital electrodes to simultaneously monitor sweat rate and ionic concentration (Fig. 10i, j) [302]. An adaptive resetting mechanism allowed continuous monitoring without manual intervention. This sensor accurately measured sweat rates (0.2–4.0 μL min^−1^) and ionic concentrations (10–200 mmol L^−1^).

Microfluidic design modifications can enhance rapid sweat absorption. A system featuring fluidic channels with chamber junctions converted continuous sweat flow into consistent-volume droplets for improved reliability [303]. A top fluidic channel collected and released sweat, while chamber junctions created discrete sweat droplets. Filter paper-lined bottom channels rapidly absorbed and evaporated droplets via capillary action (Fig. 10k, l). Hydrophobic microchannels facilitated consistent droplet formation, enabling continuous monitoring of sweat production and ionic concentrations for extended periods exceeding 7000 s.

Vertical microfluidic channels can reduce hydrodynamic resistance, optimizing sweat collection and clearance. A sensor employing truncated cone-shaped vertical channels with nanomesh electrodes and sweat-clearing structures generated electrical spike patterns indicative of sweat rate and ionic conductivity (Fig. 10m, n) [304]. This design allowed efficient sweat clearance through rapid filling and emptying, enhancing sensor sensitivity and accuracy. The event-driven data transmission approach significantly reduced energy consumption, mimicking biological neuronal responses.

The resettable operation of the sweat rate sensor involves mechanisms that manually purge collected sweat, enabling continuous device usage. One design employs elastomeric suction pumps and pinch valves to manually remove sweat, resetting the device in alignment with hydration events for sustained accuracy [305]. Sweat enters a sealed collection channel, visualized via reversible light-scattering from microstructured optical elements. When sweat exceeds 25 µL, an effervescent chemical pump ejects a sensory chemesthetic agent onto the skin. Elastomeric pinch valves utilize van der Waals forces between parallel PDMS surfaces, remaining closed until lateral strain is applied. The suction pump, featuring serpentine microchannels, generates negative pressure when stretched, facilitating sweat extraction and ejection (Fig. 10o).

Conductivity and calorimetric-based sensors face challenges from salt buildup, contamination, and corrosion, resulting in unreliable measurements. Environmental factors, like air currents and body temperature variations, also complicate accurate sweat rate and temperature assessments. Figure 10p, q illustrates a sensor system that isolates electronic components from sweat and the body, mitigating these issues [306]. A thermal actuator heats the sweat, and precision thermistors measure upstream and downstream temperature differences. The device design minimizes sensitivity to environmental influences by maintaining thermal coupling but physical isolation of electronics from sweat. A Wheatstone bridge circuit with reference thermistors further reduces environmental temperature interference.

Colorimetric sensors integrated within microfluidic channels allow immediate, in situ analysis of sweat rate without external laboratory equipment. A serpentine microchannel design was developed to track sweat progression over time, incorporating multiple microreservoirs containing colorimetric reagents (e.g., glucose, lactate, chloride, pH) (Fig. 10r) [307]. Advanced designs use capillary bursting valves for sequential sampling, facilitating temporal sweat composition analysis. Color changes within channels indicate total sweat loss and instantaneous sweat rate. Additionally, water-activated dyes (e.g., CoCl_2_) provide a straightforward visual indication of sweat levels [308].

Integrating sweat rate measurements in wearable sensors enhances personalized health monitoring, offering tailored health recommendations based on individual physiological responses. However, the development and implementation of sweat rate sensors face challenges, including variability in sweat production and composition among individuals, complicating measurement accuracy. Hygrometer-based sensors using open chamber methods are susceptible to environmental influences such as airflow, whereas closed chamber methods, although more stable, require regular vapor purging for continuous use [293]. Accurate calibration is essential to account for concentration changes dependent on flow rates, ensuring meaningful and precise biomarker interpretations. Normalizing biomarker concentrations to flow rates is crucial for obtaining physiologically relevant data.

#### Blood Pressure Sensor

Blood pressure (BP) measurement is crucial for diagnosing and managing various health conditions. Traditionally, BP is monitored through either noninvasive cuff-based methods, typically used domestically, or invasive arterial lines common in clinical settings, particularly in intensive care units. While cuff-based devices offer intermittent readings, arterial lines provide continuous data but are complex, invasive, and not suitable for patient transport. Wearable technologies have emerged as an innovative solution, offering continuous, noninvasive BP monitoring. This advancement significantly reduces the risks associated with invasive methods, aids in the detection of masked hypertension, and supports effective cardiovascular risk management strategies [309]. The subsequent sections discuss noninvasive BP measurement methods, comparing the established gold standard to emerging wearable BP technologies.

##### Continues Blood Pressure Monitoring

Traditional sphygmomanometers, due to their intermittent cuff inflation and deflation mechanism, are impractical and uncomfortable for continuous monitoring. Continuous BP monitoring, however, can greatly enhance diagnostic precision, management, and treatment of hypertension and related conditions, including diabetes mellitus (DM), potentially improving patient outcomes and reducing healthcare costs. Nonetheless, continuous monitoring technologies face challenges, including motion artifacts and external interference like ambient light and unstable skin–sensor interactions [310]. Advanced signal processing methods and the integration of accelerometers can help mitigate these issues.

##### Tonometry

Tonometry is a noninvasive technique widely used in ophthalmology for intraocular pressure measurements [311]. Clinically, radial artery tonometry has been adapted to accurately measure arterial BP by capturing the central pulse pressure (PP) waveform with a handheld device. Mild pressure is applied to partially compress the radial artery, transferring arterial pressure waves to a digital sensor for continuous recording and analysis of systolic pressure (SP) and diastolic pressure (DP) [312]. The device captures the pressure waves produced by heartbeats as they propagate along the arterial walls, enabling continuous analysis of systolic pressure (SP) and diastolic pressure (DP) [313]. Mean arterial pressure (MAP) and pulse pressure (PP) can be calculated using the formula: MAP = DP + 1/3(SP–DP) [314].

**Fig. 11:**
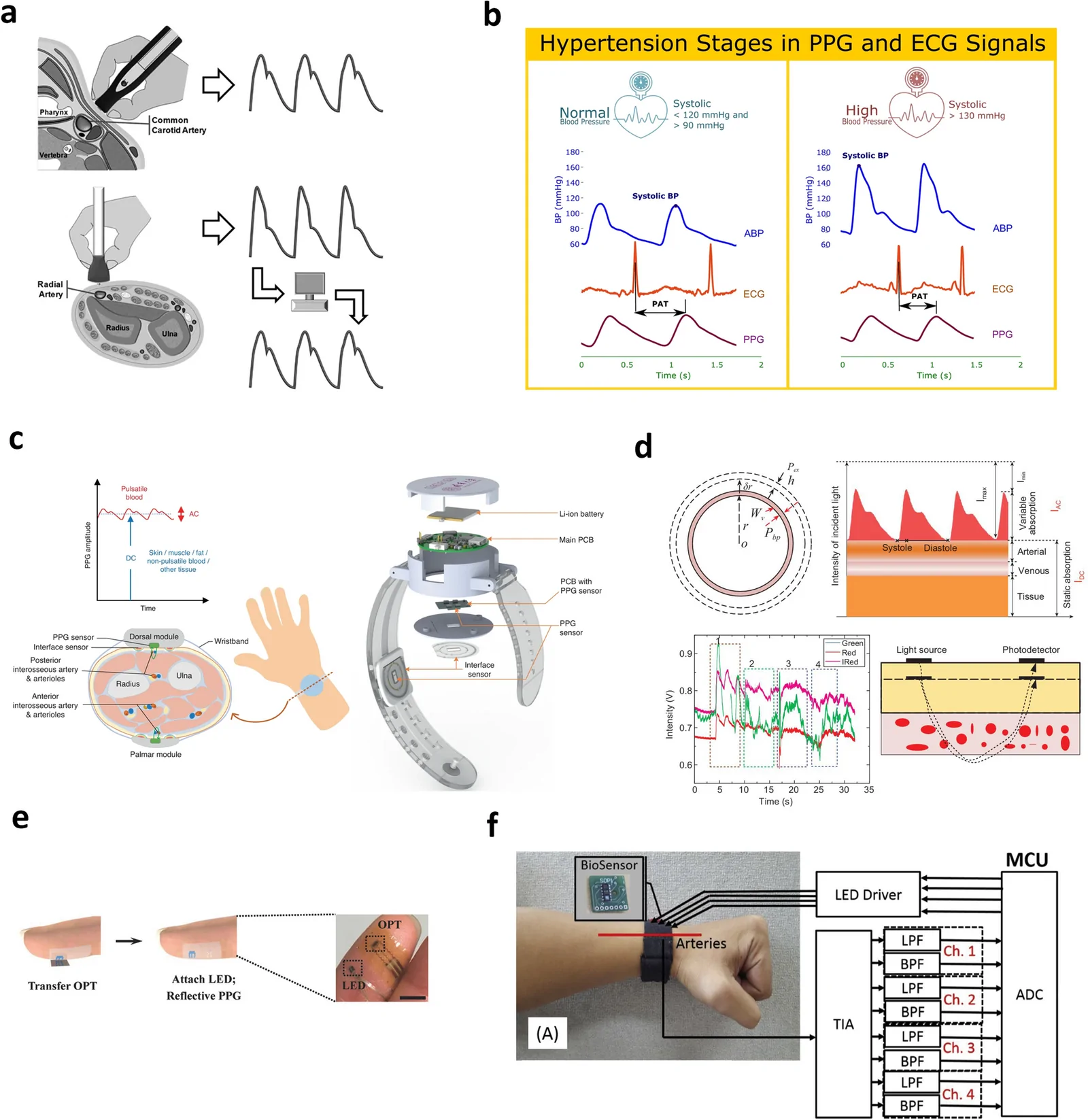
**a** Methods for assessing central blood pressure and pulse wave analysis using arterial tonometry include the "direct method" (top) and "indirect method" (bottom). Reproduced from Ref. [315] under CC BY 4.0. Copyright 2015 Salvi et al. **b** Pulse arrival time across various hypertension stages. Reproduced from Ref. [329] under CC BY 4.0. Copyright 2019 Elgendi et al. **c** Illustration of the PPG-based wristwatch. Reproduced from Ref. [331] under CC BY 4.0. Copyright 2023 Wang et al. **d** Illustration of the theoretical model for analyzing blood pressure variations using the virtual work principle (top-left). Detection of pulsating arterial blood using light transmission (top-right). Impact of motion artifacts on PPG signals and the optical path (bottom-left). Schematic of the optical path and tissue changes caused by external stress (bottom-right). Reproduced from Ref. [332] under CC BY 4.0. Copyright 2020 Li et al. **e** Illustration of the assembly process for the epidermal hybrid PPG sensor and photograph of a finger covered with the epidermal hybrid PPG sensor. Reproduced with permission from Ref. [333]. Copyright 2017 Wiley. **f** Block diagram of the proposed prototype system. Reproduced with permission from Ref. [336]. Copyright 2019 Elsevier

Tonometry-based BP measurement involves two primary methods depending on artery location (Fig. 11a). The "direct method" measures pulse waves from the accessible common carotid artery as a proxy for central aortic pressure. The "indirect method" estimates central pressure from radial artery waveforms via generalized transfer functions and linear regression models, correlating peripheral waveform features to central SP [315].

Tonometry provides significant advantages over conventional methods, which typically utilize only peripheral pulse peaks and troughs. It continuously captures detailed pulse waveforms without the discomfort of inflated cuffs [316]. Tonometry has demonstrated high accuracy and reliability in multiple studies [317].

However, tonometry has limitations. Accuracy heavily depends on precise sensor positioning, applied pressure, and signal clarity, often requiring sophisticated signal processing techniques. It struggles with high-frequency waveforms and rapid BP fluctuations [318]. Proper external pressure matching arterial transmural pressure is essential; incorrect pressure can result in inaccurate readings by either failing to couple effectively or collapsing the artery [319]. Even accurate pressure alignment can introduce errors due to inherent fluctuations between systolic and diastolic pressures [320]. Manual operation can cause misalignment issues, further affecting accuracy, necessitating calibration against standard cuff measurements [321]. To overcome these challenges, improvements, such as automated pressure adjustment systems, advanced sensor designs, sophisticated algorithms including machine learning for signal correction, and enhanced user training, have been proposed.

##### Photoplethysmography

Photoplethysmography (PPG) measures changes in blood volume within tissues over time, typically using a pulse oximeter placed on the finger. It provides a continuous, cuffless, noninvasive approach to estimate BP [322]. A PPG sensor consists of an LED light source and an optical receiver, operating either in transmission mode (detecting transmitted light through the tissue) or reflectance mode (detecting reflected light from the tissue) [323]. The underlying principle is based on infrared light absorption differences between blood and surrounding tissues, making the epidermis—rich in small vessels—an ideal measurement site [324].

Cuffless BP measurement using PPG involves two main techniques: waveform analysis and pulse transit time (PTT) [325]. Waveform analysis extracts specific features from the PPG signal, correlating these features with BP using regression models or neural networks. The PTT method, frequently preferred due to its strong physiological basis, measures the time delay between proximal and distal arterial waveforms, often using an additional sensor such as an electrocardiogram (ECG) [326]. Pulse arrival time (PAT), closely related to PTT, measures the interval between the ECG R-wave (indicating left ventricular depolarization) and a defined point in the PPG waveform. Higher BP corresponds to shorter PAT/PTT intervals due to faster arterial wave propagation, making these measurements useful for hypertension diagnosis (Fig. 11b) [327–329].

PTT specifically refers to the interval between the ECG R-peak and the corresponding peak of the PPG waveform within the same cardiac cycle. PAT includes PTT and the pre-ejection period (PEP), the interval from ventricular depolarization to aortic blood ejection [327]. Studies indicate a robust correlation between PTT and BP, supporting its use in continuous, ambulatory BP monitoring [330].

PPG-based BP measurement offers numerous advantages, including noninvasiveness, reduced patient discomfort, and minimal infection risk. Small, wearable PPG sensors comfortably allow continuous, long-term monitoring and can easily integrate into daily-use devices such as fitness trackers and smartwatches. Additionally, their low-power consumption makes them suitable for battery-operated wearable systems.

Recent research has advanced the application of PPG sensors for BP monitoring. For example, Wang et al. introduced a wearable dual-PPG wrist device incorporating contact pressure and skin temperature sensors, combined with machine learning algorithms for real-time BP estimation, significantly reducing user-related variability (Fig. 11c) [331]. While ECG alone was found less effective compared to PPG for BP estimation, it still holds potential as part of multi-sensor wearable systems. In a clinical study involving 30 healthy volunteers, BP changes induced by phenylephrine were analyzed using features extracted from PPG and ECG signals. The results demonstrated PPG’s strong potential and suitability for further development in cuffless BP monitoring [322].

Continuous blood pressure monitoring is critical for managing cardiovascular complications commonly associated with diabetes. An innovative skin-like optoelectronic system for continuous, noninvasive arterial pressure monitoring was developed, incorporating ultrathin flexible circuits and advanced theoretical models to accurately predict blood pressure and effectively suppress motion artifacts [332]. Employing a dual-channel configuration, the system reliably captured PPG signals from different locations on the body, preventing optical interference. Optical frequency-domain differentiation was utilized to stabilize measurements and reduce movement-induced noise. Clinical validation involving 44 ICU patients demonstrated high accuracy, with absolute errors of ± 7/ ± 10 mmHg for diastolic/systolic BP during stationary conditions and ± 10/ ± 14 mmHg during movement scenarios (Fig. 11d).

In related research, an epidermal flexible near-infrared PPG sensor combining an organic phototransistor and an inorganic LED was developed, enabling enhanced cardiovascular monitoring (Fig. 11e) [333]. This sensor reliably tracked heart rate variability and pulse pressure across different postures, achieving a mean absolute error below 5 mmHg compared to conventional cuff-based measurements.

ECG and PPG signals also offer promising noninvasive blood glucose (BG) monitoring capabilities essential for diabetes management. Fluctuations in BG levels affect the autonomic nervous system, altering ECG and PPG signals. A study demonstrated a spatiotemporal decision fusion strategy combining ECG and PPG signals with a Choquet integral, significantly improving BG monitoring accuracy [334]. This approach, tested with 21 participants over 103 days, achieved a root-mean-square error of 1.49 mmol L^−1^, a mean absolute relative difference of 13.42%, and placed 99.49% of results within clinically acceptable zones (A and B) on the Parkes error grid. Additionally, another study presented a noninvasive BG estimation system using wristband-based PPG data combined with physiological parameters (age, weight, height) [335].

Optical biosensors employing visible–near-infrared (Vis–NIR) spectroscopy can continuously monitor BG noninvasively. Such a system measured both pulsatile and nonpulsatile blood volume components within wrist tissues, demonstrating high correlation between estimated and reference BG values [336]. Blood viscosity in diabetic individuals influenced blood flow, reflected in PPG signal changes. Figure 11f outlines this prototype system, highlighting key components such as signal filtering, trans-impedance amplifier, LED drivers, LEDs, output channels, filters, and microcontrollers.

Despite significant advancements, noninvasive BP estimation using PPG still faces challenges in accuracy and reliability compared to traditional methods like sphygmomanometers or invasive arterial measurements [337]. Factors, such as skin tone, ambient lighting, motion artifacts, and physiological differences among individuals (e.g., age, gender, vascular anatomy), impact PPG accuracy [338, 339]. Algorithms addressing these variabilities and periodic calibration against conventional methods are essential. Although combining ECG signals or individual calibrations can enhance accuracy, these steps may reduce user convenience and portability. Large-scale validation and rigorous real-world testing are needed to establish PPG-based BP monitoring as a clinically viable tool, particularly in contexts such as diabetes management. Advancements in waveform analysis, pulse transit time techniques, system integration, device synchronization, and power management are essential for achieving widespread clinical adoption.

## Material Properties of Wearable Biosensors

Wearable sensors have specific operational needs, requiring substrate materials that provide essential flexibility, stretchability, and durability to maintain device functionality. Natural textiles such as cotton, wool, and silk are widely used due to their comfort, mechanical strength, flexibility, and established manufacturing processes like weaving [340, 341]. However, their intrinsic lack of electrical conductivity limits direct application in wearable sensors. This limitation is often overcome by applying conductive coatings, integrating metallic or carbon-based yarns, or embedding conductive polymers into the fabric [94, 342, 343].

Synthetic polymers are pivotal for wearable sensor development because they can be easily processed through methods like spinning, 3D printing, and casting. Polymers, like polydimethylsiloxane (PDMS), are biocompatible and suitable for prolonged skin contact, whereas other synthetic polymers may present risks with extended exposure, driving interest in developing eco-friendly alternatives [342]. Hydrogels, both natural and synthetic, stand out for their excellent biocompatibility, ideal for soft electrodes or microneedle arrays. However, their mechanical durability is generally insufficient for long-term wearable applications [344, 345].

Selecting materials for wearable devices involves considering multiple factors such as intended use, performance requirements, scalability, cost-effectiveness, and sustainability [84]. Material composition fundamentally determines essential wearable device properties, including flexibility, durability, and user comfort, largely influenced by the material's molecular structure [346]. Commonly used flexible substrates include silk [347, 348], cotton [349, 350], wool [351], paper [352], leather [353], vinyl [354], polyester [355], spandex [356], and polyurethane [357]. Ongoing research continues to focus on effectively integrating electronics into these substrates. Table 5 summarizes various functional materials and substrates employed in wearable sweat sensors.

**Table 5:** Properties of various materials for wearable sweat sensors

Material Class	Electrical Conductivity	Thermal Conductivity	Corrosion Resistance	Mechanical Strength	Flexibility	Stretchability	Biocompatibility	Chemical Stability	Moisture Absorption	Transparency	Durability	Biodegradability	Referencess
Metals	High (e.g., gold, silver)	High	High (e.g., gold, platinum)	High (e.g., stainless steel)	Low to moderate	Low	High (e.g., gold, platinum)	High (e.g., platinum)	Low	Low	High	Low	[94, 358–361]
Conducting Polymers	Moderate to high (e.g., PEDOT )	Moderate	Moderate	Moderate	High	Moderate	High	Moderate to high	Low to moderate	Moderate to high	Moderate	Low	[358, 359, 362–364]
Carbon-based nanomaterials	High (e.g., graphene, CNTs)	High	High	High	High	High	High	High	Low	Moderate	High	Low	[358, 359, 365–367]
Elastomers	Low	Low	Low	Moderate	High	High	High	Moderate	Moderate	Low	High	Low	[365, 368–370]
Hydrogels	Low	Low	Low	Low	High	High	High	Low to moderate	High	High	Moderate	Low	[358, 362, 371–374]
Textiles	Low	Low	Moderate	Moderate to High	High	High	High	Moderate	High	Low to moderate	Moderate	High	[94, 358, 359, 375–377]
Papers	Low	Low	Low	Low	High	High	High	Moderate	High	Low to moderate	Moderate	High	[378–381]

E-skins are integral to health monitoring due to their ability to conform comfortably to the body’s soft, curved surfaces, while meeting standards for safety, nontoxicity, and biological compatibility. Their lightweight, consistent performance, mechanical flexibility, and water resistance enable safe and prolonged integration with human tissues. This section reviews key material properties essential for wearable sweat-sensing applications.

### Biocompatibility

Wearable medical devices significantly enhance health care through real-time monitoring directly interfacing with the human body. Biocompatibility ensures that devices are safe, comfortable, and nondisruptive to daily activities. However, some active materials pose health risks upon prolonged skin exposure. For example, carbon nanotubes (CNTs) have exhibited toxicity resembling asbestos-related issues in animal studies, emphasizing the need to clearly define safe exposure limits [382].

Biocompatible organic polymers, like poly(3,4-ethylenedioxythiophene) (PEDOT) and polypyrrole (PPy), have demonstrated effectiveness in cellular monitoring applications [383, 384]. Additionally, natural and synthetic fibers and yarns from everyday textiles are being explored for creating wearable sensors [385]. Materials derived from nature, including alginate, silk fibroin, and cellulose, are increasingly popular for their biocompatibility and ease of processing [386, 387]. Cellulose, specifically, is advantageous for long-term use [382, 388], while synthetic inert materials also offer reliable biocompatibility [389]. Bioresorbable materials, capable of natural degradation, are particularly suitable for disposable and single-use sensing platforms [384, 390]. Recent research highlights conductive carbon nanofibers obtained from natural silk fibers as promising materials [391]. Additionally, various metals (e.g., titanium, gold), alloys, ceramics, polymers, and composites have been studied extensively for biocompatibility [392]. It is essential that wearable electronics are nonallergenic, nontoxic, nonirritant, and preferably biodegradable, to avoid immune reactions [393].

### Conformality and Conformability

Wearable sensing systems must effectively conform to the irregular surfaces of the human body for accurate health monitoring. Traditional rigid systems with high-power requirements often fall short in this aspect. Flexible, ultrasensitive wearable biosensors offer a superior alternative.

Reducing device thickness enhances adaptability and minimizes user discomfort, particularly for skin-mounted or implantable electronics. However, thin structures pose fabrication challenges, requiring rigid substrates for microprocessing stability. Ultrathin polymer films often necessitate sacrificial layers, removable by etching after fabrication, to facilitate their detachment [384].

Ensuring seamless contact between wearable sensors and skin involves selecting materials that match the mechanical properties of human tissues. Flexible and stretchable materials like polyimide (PI), PDMS, or poly(styrene–butadiene–styrene) (SBS) are commonly employed [394]. Conductive nanomaterials form durable, flexible inks and connection circuits, carefully balancing conductivity and stretchability through material selection and design patterns such as mesh or serpentine configurations [394, 395]. Despite these advancements, challenges persist in developing ultrathin, flexible sensors. Key issues include refining fabrication methods, ensuring efficient detachment processes, and precisely balancing mechanical flexibility with electrical performance.

### Flexibility

The evolution of wearable biosensors demands substrates with enhanced flexibility and minimal surface roughness, replacing traditional rigid materials such as glass, silicon, or SiO_2_. Flexible substrates ensure seamless adaptability to various physiological surfaces, critical for effective wearable technology. Rubber and elastic polymers are preferred due to their elasticity, chemical resistance, and thermal stability. PDMS stands out for its exceptional flexibility, hydrophobic nature, and high tensile strength (up to 1000%), making it ideal for various wearable applications [396]. Soft lithography techniques allow intricate microstructures to be formed on PDMS substrates, although challenges related to long-term stability and thermal performance persist [384, 397, 398]. Other polymers such as poly(ethylene naphthalate) (PEN), polyethylene terephthalate (PET), polyvinyl alcohol (PVA), and PI are also commonly utilized. PI is notable for chemical and thermal resilience [399], while PEN and PET are preferred for their optical transparency despite lower thermal stability compared to PI [368].

Flexibility can be further improved by modifying molecular configurations, such as extending side chains to reduce rigidity [400, 401], or using strategies to decrease van der Waals interactions through intentional fracturing or mechanical buckling [402]. Incorporating plasticizers, cross-linkers, and dissipative materials like alginate, hyaluronan, and chitosan can enhance flexibility and adjust the elastic modulus [403, 404]. Decreasing entanglement density also contributes to flexibility and stretchability [405]. Sensors featuring flexible designs, such as fractal or serpentine structures, effectively reduce tensile strain and enhance mechanical compatibility with biological tissues [406, 407].

### Adhesion

Adhesion significantly influences the effectiveness, user comfort, and reliability of wearable biosensors, particularly those interfacing with skin. Reliable adhesion ensures stable signal transmission, comfort during use, and ease of removal. Traditional adhesive methods, including medical tapes and bandages, are effective but may leave residues and cause discomfort upon removal.

The adhesion quality depends on the surface energy of both skin and sensor materials; materials with high surface energy typically exhibit superior adhesion. Ultra-lightweight, thin electrode tattoo patches utilize surface tension for gentle skin attachment. However, persistent challenges, particularly on sweaty skin, have encouraged research into dry adhesive electrodes providing robust yet easily removable adhesion [408, 409]. Hydrophilic adhesives capable of maintaining stickiness in moist conditions are particularly useful for sweat-monitoring sensors.

Biomedical patches used for medical treatments as scaffolds or matrices also face challenges related to device breakdown and allergic reactions. Medical adhesives like acrylics can cause allergic contact dermatitis from prolonged skin exposure [410, 411]. Difficulty detaching devices from skin further emphasizes the need for advanced adhesion solutions that balance strong attachment with easy removal. Patterned adhesives have been developed to improve breathability, reduce irritation, and maintain adhesion by facilitating moisture release.

Degradable polymers, such as polylactic-*co*-glycolic acid (PLGA), chitosan, gelatin, collagen, polycaprolactone (PCL), and polylactic acid (PLA), have been studied extensively as biosensor patch materials due to their cost-effectiveness and favorable mechanical properties [323, 411, 412]. However, natural biopolymers often display limited mechanical robustness and hydrophobic surface characteristics, restricting their broader use [411].

Ongoing innovations in adhesion technologies for wearable biosensors continue to address these challenges, enhancing their effectiveness, comfort, and user-friendliness. Although regulatory hurdles and clinician hesitancy remain obstacles, advancements in this field steadily drive wearable sensor technologies toward broader acceptance and commercialization.

### Self-Healing

Wearable devices are susceptible to mechanical damage, which can compromise their functionality and sensitivity. To address this, research has focused on developing self-healing materials capable of restoring mechanical and electrical properties after minor damages. Prominent self-healing materials include composites embedded with healing agents or conductive particles, emulating the natural regenerative properties of human skin [384]. Materials employing dynamic interactions and reversible chemical bonds have proven effective in both dry and wet conditions, offering robust self-repair capabilities [413].

The soft nature of wearable sensors, crucial for accurate biological signal detection and comfort, inherently increases susceptibility to mechanical damage. Integrating self-healing properties significantly improves their durability and reliability, particularly in demanding environments. Intrinsic polymers utilize reversible bonds, while extrinsic polymers incorporate healing agents encapsulated within their structures [414]. Such self-healing innovations enhance sensor reliability, longevity, and cost-efficiency for precise in vivo diagnostics.

Stability and resilience of medical devices remain critical. Self-healing flexible sensors have demonstrated the ability to autonomously repair internal or external damages, restoring essential functionalities Materials capable of regaining their mechanical strength and shock resistance after damage are particularly valuable during active human use. Recent advances, including reconfigurable liquid–metal electrodes and fully self-healing electronic frameworks, showcase promising developments in self-repairing electronics [415].

### Other Properties

Wearable sensor effectiveness significantly depends on permeability, enhancing comfort and suitability for long-term wear. Early wearable designs utilizing rigid materials like PI or PDMS lacked sufficient gas and moisture permeability [416]. More recent innovations have introduced breathable substrates such as textiles and ultrathin, freestanding tattoo-like electrodes, allowing better heat, air, and moisture transfer. However, high permeability may compromise the accuracy of sweat-based biomarker detection. To overcome this, nanomesh structures with very small pores facilitate gas permeability while preventing sweat infiltration, improving measurement reliability [417].

Electrical conductivity is another essential characteristic of wearable biosensors, requiring stable performance under mechanical strain and exposure to sweat. Conductive polymers and hydrogels have emerged as promising alternatives, offering flexibility, cost-effectiveness, and reliable conductivity [418, 419]. The inclusion of conductive nanomaterials, such as nanoparticles and nanowires, further enhances sensor conductivity.

Transitioning from laboratory prototypes to practical, large-scale applications demands cost-effective and scalable manufacturing methods. While traditional lithography techniques are expensive [420], alternative methods, including transfer printing [421], electrospinning [422], roll-to-roll gravure printing [423], laser engraving [295], 3D printing [424], inkjet printing [425], screen printing [426], wet spinning [427], and embroidery [428], have emerged as viable solutions tailored to the specific requirements and material properties of wearable sensors.

To ensure effective functionality, wearable biosensor materials must possess biocompatibility, electrical conductivity, user comfort, and scalability. For sweat-sensing applications, additional criteria like robust adhesion to sweaty skin, durability in humid conditions, and selective permeability to gas and sweat are crucial. Ongoing advancements in material science and engineering continuously enhance wearable biosensors, increasingly meeting the diverse and evolving needs of personal health monitoring, particularly for diabetes management.

## Multimodal Sensors

Wearable sensors have progressed from basic devices measuring single parameters to sophisticated platforms capable of simultaneously monitoring multiple biomarkers and biosignals. Multiplexed sensing is particularly beneficial for managing Type 1 Diabetes (T1D), offering crucial data for personalizing insulin dosages and understanding relationships between biomarkers under various physiological conditions. Integrating multiple sensing modalities allows real-time tracking of diabetes-related biomarkers, such as glucose, branched-chain amino acids (BCAAs), and lactate, alongside biosignals including blood pressure, sweat rate, pH, electrocardiogram (ECG), and temperature. This comprehensive approach is vital for effective diabetes management and early detection of complications.

**Fig. 12:**
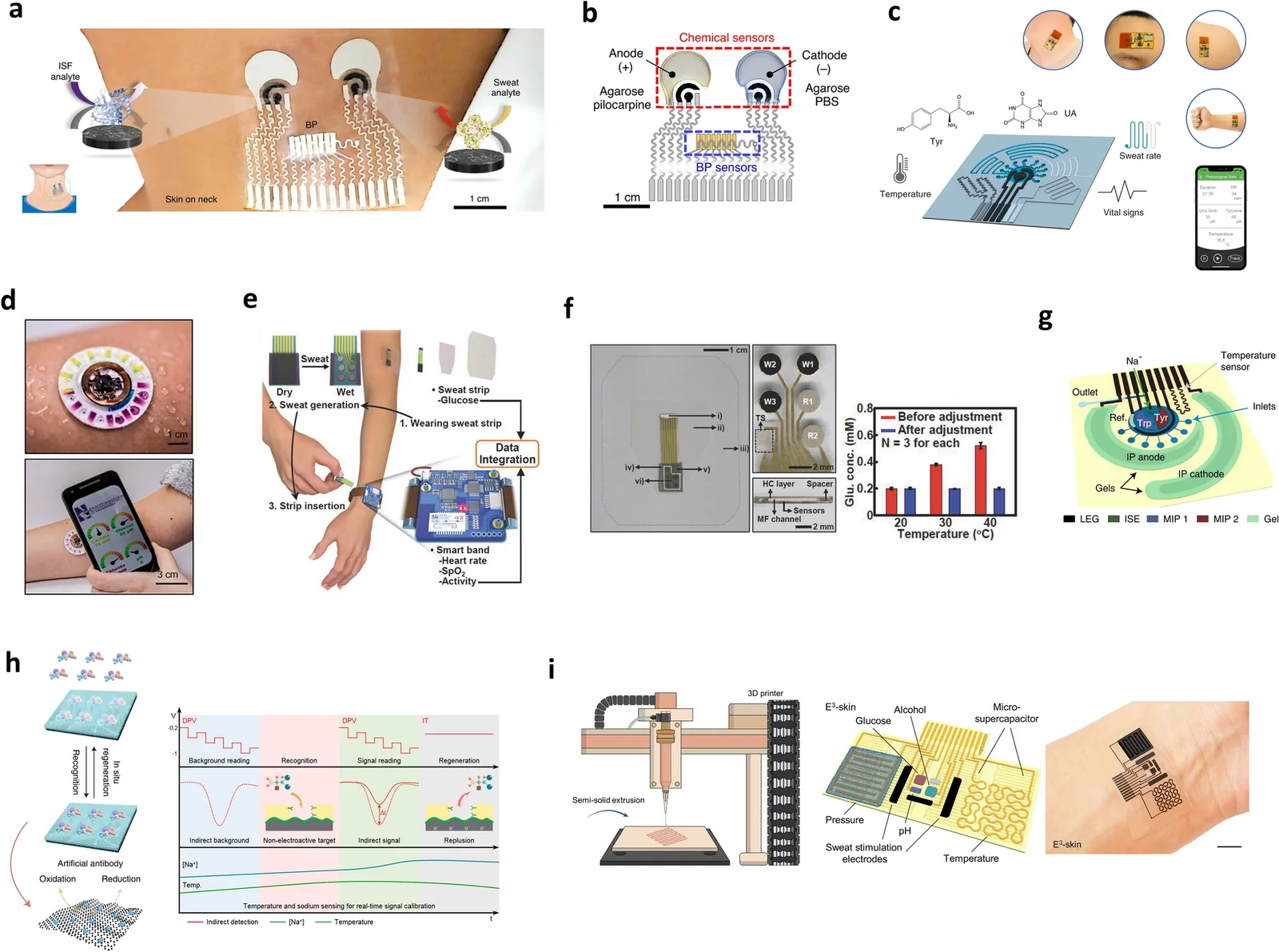
Multimodal sensor. **a** Illustrations of the acoustic sensor and the enzymatic chemical sensors for ISF and sweat. **b** Illustration of the acoustic and electrochemical sensing components along with hydrogels for sweat stimulation and ISF extraction. Reproduced from Ref. [429] under CC BY 4.0. Copyright 2021 Sempionatto et al. **c** Multiple sensor functions, such as ultrasensitive detection of UA and Tyr, sweat rate estimation, temperature sensing, and vital sign monitoring. Photographs of the sensor worn on different body parts. Reproduced with permission from Ref. [295]. Copyright 2019 Springer Nature. **d** Illustration of the device during sweating and a phone interface. Reproduced from Ref. [433] under CC BY 4.0. Copyright 2019 Bandodkar et al. **e** An overview of the wearable health management system consists of a disposable sweat-analysis strip for glucose measurement and a wearable smart band for monitoring heart rate, blood oxygen saturation level, and physical activity. **f** Integration of the glucose and temperature sensors, and sweat glucose levels at different temperatures before and after adjusting for temperature dependency of the glucose sensor. Reproduced with permission from Ref. [434]. Copyright 2018 Wiley. **g** Illustration of the multifunctional wearable sensor. **h** In situ regeneration and calibration technologies of the sensor. In situ calibration strategies of the molecularly imprinted polymer (MIP)-redox-active reporter (RAR)- laser-engraved graphene (LEG) sensor with indirect detection mechanism during on-body use. Reproduced with permission from Ref. [76]. Copyright 2022 Springer Nature. **i** Schematic illustration of the SSE-based 3D printing and the 3D-printed sensor and optical images of the sensor. Reproduced from Ref. [437] under CC BY 4.0. Copyright 2023 Song et al.

Advanced epidermal patches enable simultaneous monitoring of biomarkers and cardiovascular parameters in both ISF and sweat. For example, a patch combining ultrasonic transducers and electrochemical sensors measured blood pressure (BP), heart rate (HR), glucose in ISF, and sweat biomarkers like lactate, caffeine, and alcohol (Fig. 12a, b) [429]. The ultrasonic transducers emitted pulses reflected by arterial walls, measuring echo times correlated with artery dynamics, thus providing accurate BP and HR readings.

Skin-interfaced multimodal sensors integrate biochemical and biophysical sensing in flexible, wearable formats suitable for continuous health monitoring, from daily tracking to clinical diagnostics. A laser-engraved wearable sensor exemplifies this by detecting sweat biomarkers such as uric acid (UA) and tyrosine (Tyr), alongside vital signs like temperature and respiration rate (Fig. 12c) [295]. Its microfluidic module dynamically managed sweat sampling, minimizing contamination and evaporation, offering precise metabolic state insights, including early detection of gout or metabolic disorders.

Combining electrochemical and optical sensing facilitates simultaneous chemical detection and identification. Dual-mode sensing arrays integrating enzyme-based amperometric sensors and ion-selective electrodes alongside colorimetric sensing arrays have been developed to concurrently monitor glucose, lactate, pH, chloride, and urea, using functionalized nanofiber films for visual biomarker detection [430].

Multimodal sensing provides a comprehensive health assessment, enabling early identification of complications like ketoacidosis or hypoglycemia. For instance, a sudden glucose fluctuation combined with abnormal ECG signals could indicate cardiovascular issues, necessitating immediate intervention. The Janus on-skin wearable simultaneously tracks electrical signals from the heart (ECG), brain (EEG), and muscles (EMG), along with biochemical markers such as glucose, uric acid, and caffeine. Environmental factors, like UV intensity, humidity, ammonia, and alcohol presence, were also measured, enhancing diagnostic accuracy by cross-validating data to minimize false alarms [431].

Integrating diverse sensing technologies—immunoassays, fluorescence assays, impedance measurements, and electrochemical sensors—allows comprehensive physiological and environmental monitoring. For instance, a wearable device combined cortisol immunoassays, fluorescence-based glucose and ascorbic acid detection, galvanic skin response (GSR) measurements, and electrochemical sensors for sweat conductivity and production rate, all powered wirelessly via near-field communication (NFC) [432]. Another innovation combined electrochemical sensors and colorimetric assays within microfluidic channels, simultaneously assessing lactate, glucose, chloride, and pH, with sweat rate visually tracked through dye-based color changes (Fig. 12d) [433]. This hybrid design merges electronic and visual analytics, improving sensor versatility and user convenience.

Multimodal sensing offers significant advantages, particularly in enhancing the reliability and accuracy of health data. Combining various sensor types allows for cross-validation, reducing false readings and ensuring precise measurements, crucial for informed diabetes management decisions. For instance, an epidermal patch based on nanoporous carbon-MXene heterostructured nanocomposites simultaneously monitored glucose, lactate, pH, temperature, and electrophysiological signals on a flexible PET substrate​ [228]. Discrepancies in glucose readings were cross-referenced against lactate and ECG data to distinguish actual glucose fluctuations from stress-induced anomalies. Real-time calibration mechanisms for temperature and pH variations ensured accurate glucose measurements in sweat.

A significant challenge with multimodal sensors is cross-talk and interference among different sensing modalities. Minimizing such interference involves spatially separating sensor electrodes or employing hydrophobic isolation layers. For example, a hydrophobic layer effectively separated lactate and ECG sensors, increasing impedance to minimize signal distortion from sweat and ensuring accurate simultaneous measurements [426]. This sensor system included a potentiostat for lactate detection, an ECG analogue front-end, and Bluetooth communication for wireless data transmission.

Enzyme-based sensors are sensitive to temperature fluctuations, necessitating integration with temperature sensors or employing calibration curves that adjust biomarker concentrations accordingly. A wearable device combined biochemical sensing (glucose) with physiological monitoring (heart rate, SpO_2_, physical activity) to manage diabetes and prevent hypoglycemia during exercise (Fig. 12e, f) [434]. Real-time temperature monitoring within the sweat-analysis strip allowed temperature-adjusted glucose readings through calibration, ensuring accuracy despite environmental variations.

Similarly, pH variations influence chemical sensing accuracy in sweat analysis. A biofuel-powered electronic skin integrated sensors for glucose, urea, ammonium, pH, and temperature on a unified platform, featuring onboard calibration for both pH and temperature variations [435]. Real-time pH measurement enabled dynamic adjustment of chemical sensor readings, with additional calibration ensuring linear responses across analyte concentration ranges.

Despite advances, multimodal wearable devices face challenges in efficiently integrating and interpreting diverse sensor data. Robust data processing tools and machine learning algorithms are essential to handle substantial data volumes, synchronize multiple sensor inputs accurately, and interpret complex physiological interactions. Advanced machine learning techniques are particularly valuable for developing predictive models capable of personalized health insights and early complication detection.

Integrating in situ regeneration and calibration technologies further enhances wearable biosensor capabilities. Sensors depicted in Fig. 12g, h demonstrate regeneration via high-voltage amperometry, oxidizing and removing bound target molecules to reset sensor functionality without external intervention [76]. These sensors, employing laser-engraved graphene (LEG), redox-active nanoreporters, and molecularly imprinted polymers (MIPs), measured biomarkers like amino acids, vitamins, metabolites, and electrolytes alongside vital signs such as temperature. Real-time calibration utilized integrated temperature and ion-selective Na⁺ sensors, accommodating variations in sweat composition and environmental conditions. For instance, LEG-MIP sensors employed a two-scan DPV method for tryptophan detection, differentiating baseline and bound-state oxidation peaks for improved measurement accuracy (Fig. 12h).

Real-time data transmission to external devices like smartphones or cloud servers is crucial for timely analysis and medical intervention. Wearable devices typically employ low-power microcontrollers and efficient communication protocols such as Bluetooth low energy (BLE) or near-field communication (NFC) to manage data effectively [60]. Effective power management strategies, including energy harvesting techniques, help extend device usage without frequent recharging.

Microfabrication and additive manufacturing advancements have significantly improved the miniaturization and integration of multiple sensing modalities into wearable devices. Techniques, such as photolithography, thin-film deposition, and microfluidics enable, compact, flexible sensor designs suitable for comfortable patient wear [436]. Additive manufacturing methods, particularly 3D printing, have facilitated cost-effective and precise sensor production. For example, Fig. 12i illustrates a 3D-printed epifluidic electronic skin sensor capable of monitoring glucose, alcohol, pH, heart rate, and temperature [437]. Using semisolid extrusion-based 3D printing technology, researchers precisely patterned multidimensional nanomaterials, polymers, and hydrogels onto flexible substrates, enhancing the sensor’s functionality and user comfort.

Closed-loop diabetes management systems combine continuous monitoring and therapeutic intervention to offer holistic disease management. Integrating therapeutic components enables immediate responses to hyperglycemic events, significantly improving patient care [438, 439]. One integrated system included a sweat-uptake layer, waterproof film, and sensors for humidity, glucose, pH, and tremors, alongside microneedles thermally activated to deliver drugs such as Metformin transcutaneously [439]. The microneedles were coated with a bioresorbable polymer and phase-change material that melted at specific temperatures, allowing controlled drug release based on glucose measurements. Such integrated monitoring-therapy devices reduce the burden of constant glucose checks and medication administration, significantly improving patient compliance and convenience.

In addition to sweat, incorporating multiplex detection from alternative biofluids, such as interstitial fluid (ISF) and tears, can significantly enhance the validity and clinical reliability of noninvasive biomarker analysis. Since individual biofluids differ in composition, biomarker concentration dynamics, and temporal response, simultaneous monitoring across multiple compartments offers a more comprehensive physiological snapshot. For example, coupling sweat-based glucose sensing with ISF-derived glucose measurements can help cross-validate readings, reduce signal variability, and improve calibration accuracy under dynamic conditions. Similarly, tear fluid—accessible via smart contact lenses—offers potential for continuous glucose monitoring with minimal discomfort.

Advances in materials science and flexible electronics will further enhance wearable device comfort and unobtrusiveness, improving patient adherence and enabling continuous everyday health monitoring. Developing ultrathin, flexible sensors integrated into clothing or directly adhered to skin will significantly improve usability. Future wearable sensors may include an expanded array of biomarkers, such as cytokines and other inflammatory markers, providing deeper insights into immune responses and inflammation in diabetes patients. Comprehensive biomarker detection can facilitate early diagnosis of complications and provide a detailed understanding of disease progression.

Future research should address challenges such as accurate sensor calibration under varying physiological conditions. Although current efforts integrate sensors for pH, temperature, and sweat rate, additional refinement is required to enhance measurement accuracy. Large-scale clinical trials are necessary to validate system efficacy in practical settings and explore the clinical significance of emerging biomarkers.

To ensure accurate interpretation of multimodal data streams, wearable platforms increasingly rely on dynamic calibration strategies. Integrated pH, temperature, and sweat rate sensors correct biochemical readouts in real-time, while machine learning algorithms adapt to user-specific physiological baselines and environmental variations. These adaptive systems, supported by multi-sensor fusion and pattern recognition models, enable robust calibration across diverse conditions and individual differences, improving the clinical utility of wearable diabetes monitoring tools.

The future of wearable multimodal sensing in diabetes care lies in personalized healthcare. Advanced data analytics and machine learning algorithms can deliver tailored recommendations and insights, significantly enhancing patient outcomes and improving quality of life for individuals with diabetes.

## Challenges and Future Direction

### Challenges

**Fig. 13:**
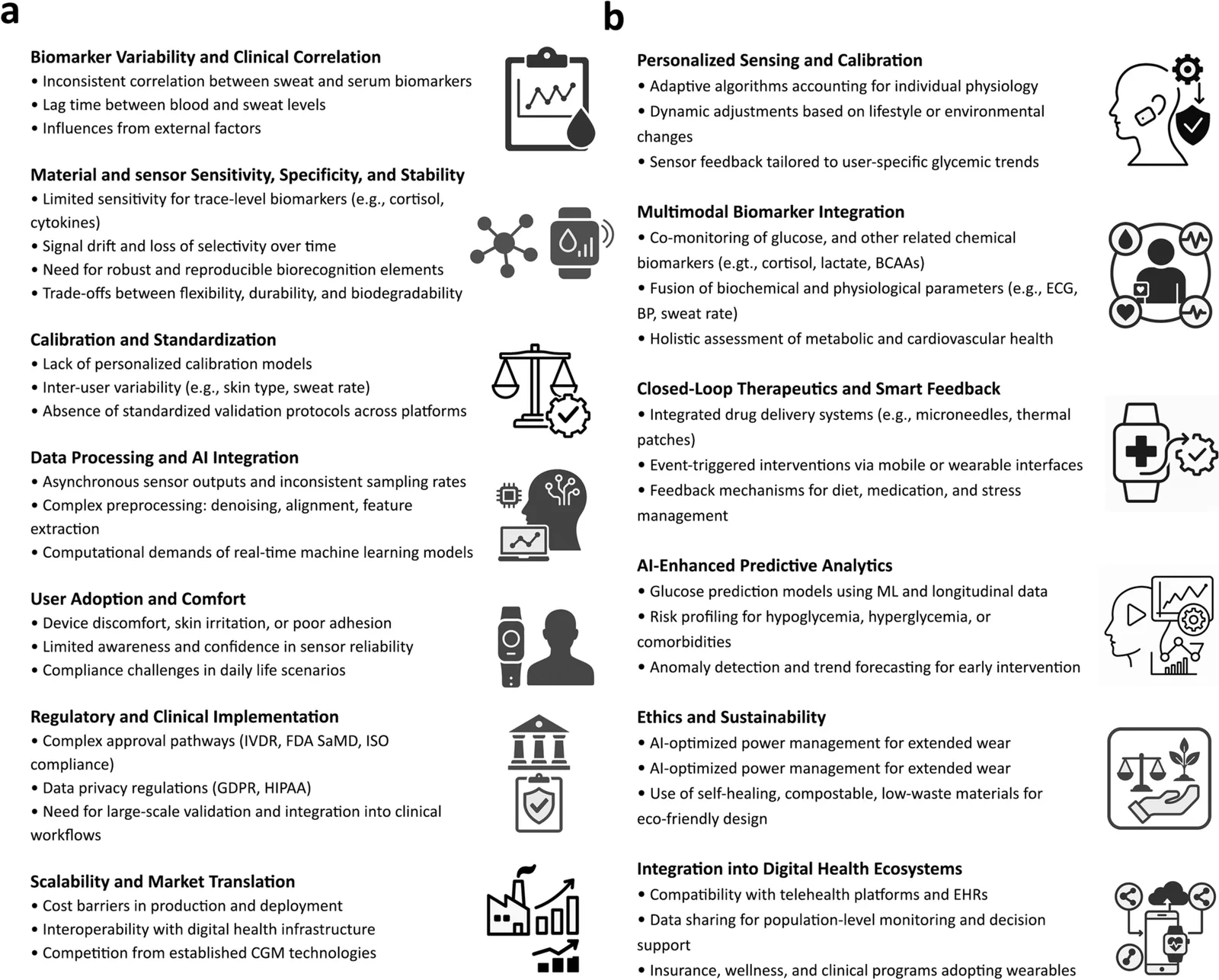
**a** Challenges and **b** perspectives for noninvasive on-skin biosensors in diabetes monitoring

Figure 13 provides a concise overview of the key challenges and future directions discussed in this section, highlighting critical areas for ongoing research and development in wearable biosensors for diabetes monitoring. Accurately understanding the relationship between biomarkers in sweat and blood serum is fundamental for effective diabetes diagnosis and monitoring. Extensive research is needed to precisely map these relationships, validate sweat biomarkers, and account for dynamic variations in sweat composition, especially concerning glucose. Investigating lag times between serum and sweat biomarker concentrations is critical for developing real-time predictive algorithms capable of forecasting blood glucose fluctuations based on sweat analysis. Variables, such as temperature, humidity, physical activity, and individual physiological differences, significantly influence sweat secretion rates, underscoring the necessity for standardized methodologies in data collection and analysis.

Cross-sensor calibration and accuracy validation present additional complexities due to variations in skin thickness, body fat composition, and user movement, all of which affect sensor performance. Furthermore, establishing standardized validation metrics to ensure clinical reliability remains challenging. User adoption also faces barriers, such as discomfort or inconsistent device usage, highlighting the need for improved design and user education to foster regular compliance.

Future wearable devices should aim to integrate biochemical markers such as glucose, cortisol, BCAAs, lactate, and cytokines, alongside physiological markers including heart rate, blood pressure, and ECG signals. Monitoring a comprehensive spectrum of biomarkers will provide deeper insights into metabolic and physiological health, greatly enhancing personalized diabetes management strategies. Understanding interrelationships among these biomarkers will improve precision in monitoring and offer insights into how lifestyle factors such as diet, stress, and exercise influence glucose metabolism and overall health outcomes.

Enhancing biosensor sensitivity, selectivity, and long-term stability remains a critical challenge. Calibration algorithms must accommodate individual variability and real-time physiological fluctuations to ensure accurate measurements under diverse conditions. While existing sensors reliably detect high-concentration metabolites, accurate measurement of trace-level biomarkers like hormones requires advances in sensor technologies and novel biorecognition materials. Demonstrating robustness and stability through extensive clinical trials and validation studies in real-world scenarios is essential.

Designing compact, reliable, and comfortable wearable devices that continuously collect adequate sweat samples poses substantial challenges. Improvements in microfluidic designs and sweat extraction methods are required to address issues related to low sweat volumes and significant interpersonal variability. Developing systems that integrate sensor arrays with energy-efficient electronics and comfortable form factors is essential to improve user compliance and enable effective health monitoring.

Additionally, data collected from various sensors often differ significantly in format, resolution, and sampling rates, complicating data management. Key technical challenges include synchronizing multi-source data acquisition, filtering noise, calibrating and aligning data for consistency, preventing data loss, optimizing data storage, and ensuring accurate real-time data processing with low latency. Leveraging advanced machine learning and artificial intelligence (AI) algorithms is necessary for deriving meaningful health insights from complex multimodal datasets.

Future research should prioritize the development of advanced, flexible, biocompatible materials, including organic semiconductors, flexible electronics, and biodegradable substrates, to enhance wearable biosensors’ performance and sustainability. Innovations in flexible battery technologies and energy harvesting techniques, such as capturing energy from body movements or ambient environments, are essential to ensure uninterrupted operation and user convenience. Utilizing environmentally friendly materials, such as biodegradable polymers and compostable cellulose substrates, is increasingly important for sustainable mass production and reducing environmental impact.

Improving user comfort, adherence, and long-term stability is central to the clinical viability of wearable biosensors. Recent strategies focus on integrating ultrathin, breathable, stretchable and biocompatible substrates—such as nanomesh textiles, hydrogels, and silk fibroin—into conformal designs that minimize irritation and thermal discomfort during extended wear. Adhesion has been enhanced through patterned dry adhesives, hydrogel interfaces, and elastomeric layers that maintain contact even on sweaty or mobile skin surfaces. For long-term durability, the use of self-healing materials, stretchable serpentine interconnects, and conformal electronics reduces mechanical fatigue and preserves signal integrity during motion.

Physiological variability across age, skin type, and physical activity levels can influence the performance of wearable biosensors. For example, older adults often exhibit reduced sweat gland density and altered skin hydration, which may impair analyte secretion and skin–sensor adhesion. Skin pigmentation affects optical sensor accuracy due to variable melanin absorption, while intense physical activity can induce motion artifacts and excessive sweat that compromise signal quality. To address these challenges, current devices leverage flexible substrates, hydrogel buffering layers, and multimodal calibration (e.g., pH, temperature, sweat rate) to normalize outputs. In parallel, machine learning models trained on population-diverse datasets enable real-time adaptation and personalized interpretation of biosignals, enhancing accuracy and inclusivity across user groups.

To overcome the limitations of conventional power supplies, self-powered and energy-autonomous systems are increasingly integrated into wearable diabetes monitoring platforms. Recent advances highlight the development of sophisticated wearable microgrid systems that combine multiple bioenergy harvesting mechanisms, such as enzymatic sweat-driven biofuel cells (BFCs) and hybrid energy harvesters. For instance, fingertip-mounted microgrid systems using enzymatic BFCs paired with stretchable AgCl–Zn batteries efficiently harvest biochemical energy from lactate and glucose in sweat, providing continuous power supply for autonomous sensing of multiple metabolites including glucose, lactate, and levodopa [10]. These microgrid systems utilize osmosis-based sweat extraction integrated with low-power electronics, ensuring stable bioenergy harvesting during daily activities and rest periods. Similarly, flexible, textile-based e-textile microgrid platforms integrate enzymatic BFCs with triboelectric generators (TEGs) to synergistically harvest biochemical and biomechanical energies [11]. Such configurations efficiently regulate and store harvested energy using supercapacitors, thereby supporting both low- and high-power wearable electronic applications, improving the sustainability and practicality of continuous monitoring systems.

**Fig. 14:**
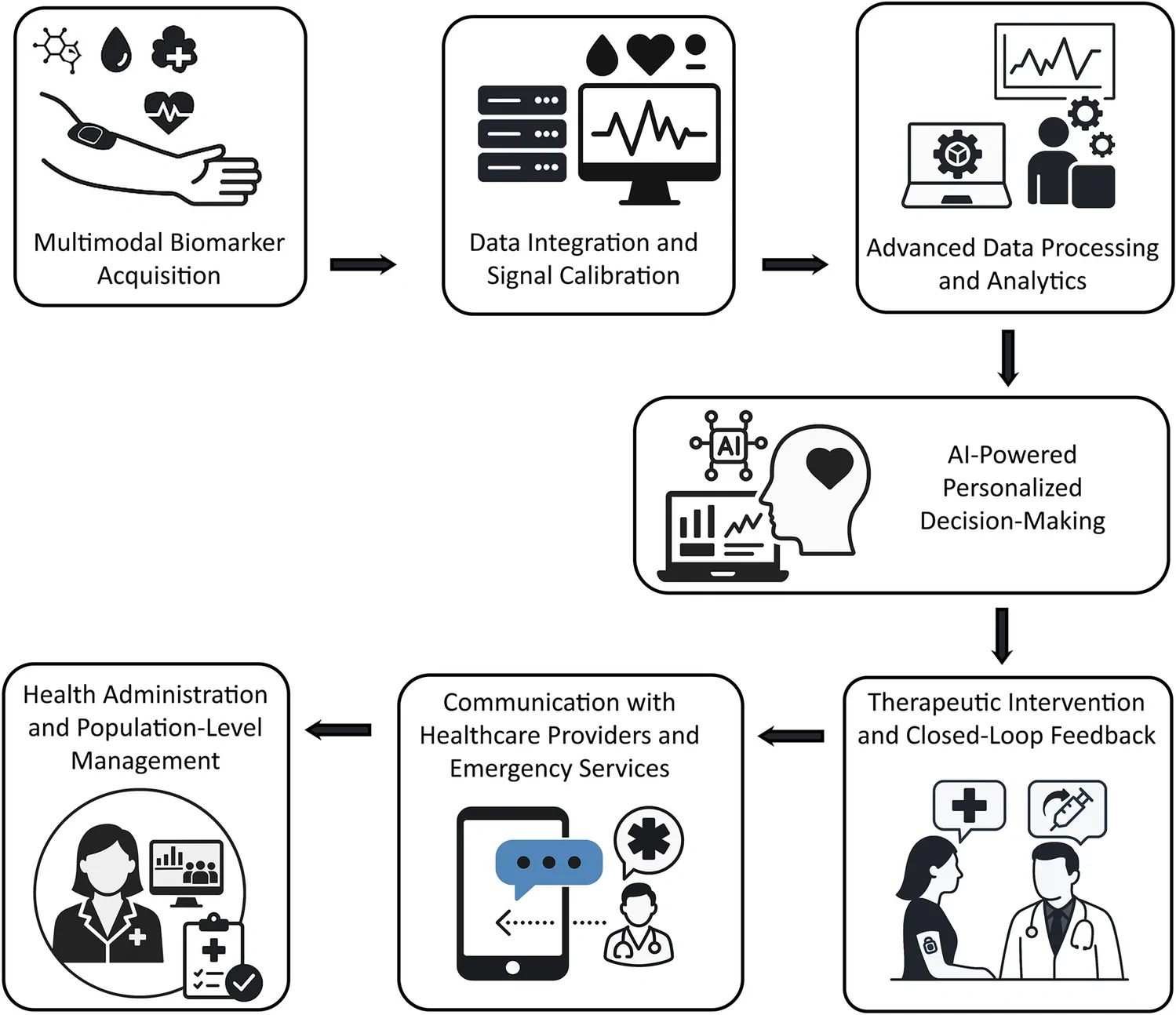
Future of diabetes management via skin-interface biosensors. Conceptual framework illustrating the envisioned pathway for future diabetes management using skin-interfaced biosensors, from real-time sensing to actionable outcomes across personal and population health levels

Alongside these harvesting innovations, system-level integration and energy optimization are essential to achieving fully autonomous sensor operation. Integrated microgrids employing biofuel cells and hybrid harvesters not only address battery-life issues but also enable real-time multiplexed biomarker sensing through complementary energy management strategies. The inclusion of supercapacitors and flexible battery modules with matching potentials minimizes energy loss, enhances reliability, and significantly reduces device footprint, ultimately improving user convenience. Future developments that focus on modular architectures, advanced low-power communication protocols, and adaptive power management circuits will further push the boundaries of wearable autonomous health monitoring platforms, facilitating reliable, sustainable, and personalized diabetes care. Figure 14 illustrates a conceptual framework for future diabetes management using skin-interfaced biosensors.

### AI, Data Analytics, and Predictive Algorithms

The integration of big data analytics and machine learning algorithms is pivotal for managing and interpreting the vast data generated by multimodal wearable biosensors. Leveraging these advanced analytics can reveal subtle correlations among biomarkers, enabling personalized predictive modeling and facilitating early detection of diabetes-related complications. Sophisticated algorithms are required to effectively synchronize, integrate, and interpret data streams from multiple sensors, providing accurate, real-time feedback and actionable insights for both users and healthcare providers.

Incorporating AI-driven analytics with wearable sweat sensors can greatly enhance continuous glucose profiling, enabling predictive capabilities for improved glycemic control. Machine learning algorithms can process real-time biosensor data to detect glucose variability patterns, thus facilitating timely interventions before hypoglycemic or hyperglycemic events occur. These advanced analytics support real-time health monitoring through IoT-enabled wearable devices, employing techniques such as pattern recognition for disease prediction, feature extraction, clustering, and anomaly detection. Integration with next-generation communication technologies, such as 5G and 6G, coupled with cloud-based platforms, significantly improves remote monitoring and immediate response capacities.

### Theranostic Applications and Closed-Loop Systems

The integration of therapeutic functionalities within wearable biosensors represents a major advancement toward automated and personalized diabetes management. Future developments may integrate continuous monitoring with responsive drug delivery systems, including microneedle arrays or smart bandages, enabling real-time therapeutic interventions triggered by biomarker fluctuations. Establishing robust closed-loop systems capable of autonomously monitoring, analyzing, and treating diabetes in real-time could greatly reduce patient burden and enhance overall disease management efficacy.

Smartphone applications integrated with wearable sensors can further refine personalized digital therapeutics for diabetes care. For example, upon detecting a potential hypoglycemic event, interconnected applications could promptly provide dietary recommendations, alert caregivers, or initiate automated glucagon release through closed-loop insulin pumps. Similarly, in cases of stress-induced hyperglycemia, applications might suggest guided relaxation techniques or adaptive insulin dosing strategies tailored to real-time physiological responses.

Furthermore, sweat-based biosensors offer potential for monitoring medication adherence and metabolic responses in patients using insulin or oral antidiabetic medications. Continuous analysis of biomarkers, such as glucose, lactate, and BCAAs in sweat, can deliver valuable feedback regarding the effectiveness of pharmacological treatments. Machine learning algorithms can enhance therapy personalization by identifying individual glucose response patterns related to medication intake, exercise, or dietary habits, thereby optimizing treatment outcomes for each patient.

Optimizing ML algorithms for wearable multimodal biosensors requires robust data pipelines that accommodate diverse sampling rates, signal types, and noise levels. Techniques, such as supervised learning, adaptive baselining, and federated learning, allow for real-time, privacy-preserving model refinement across users. Integration with mobile and cloud platforms supports scalable, individualized glycemic risk prediction, enhancing both clinical decision-making and patient self-management.

### Social Acceptance, Privacy, and Ethical Considerations

The widespread adoption of wearable biosensors extends beyond technological advancement, significantly depending on social acceptance, ethical frameworks, and rigorous data privacy protections. Educating users about the advantages, limitations, and data-handling practices associated with wearable sensors is crucial for building user trust and encouraging consistent usage. Implementing robust security protocols and complying with regulatory standards are essential to address data privacy, confidentiality, and cybersecurity concerns effectively.

Integration of wearable biosensors within broader healthcare ecosystems, such as health insurance schemes, telemedicine platforms, and healthcare provider networks, can substantially enhance their utility and encourage wider adoption. Successful implementation of these technologies in diabetes management requires collaboration among diverse stakeholders, including patients, caregivers, healthcare professionals, regulatory bodies, insurers, technology developers, and academic researchers. As healthcare shifts toward patient-centered digital tools, individuals play increasingly active roles in managing their health through personalized real-time monitoring. While this shift offers significant potential, it also presents challenges concerning user acceptance, usability, and compliance.

Meaningful patient adherence and inclusion necessitate the co-design of wearable biosensors with individuals who live with diabetes, ensuring that devices align with real-world preferences, needs, and expectations. Practical considerations, such as ease of use, reliability, comfort, and minimal disruption to daily routines, are essential to fostering patient adherence and satisfaction.

Digital biomarkers are revolutionizing diabetes management and clinical research through continuous, remote health monitoring. The Clinical Trials Transformation Initiative (CTTI), in collaboration with regulatory authorities and academic institutions, has emphasized essential guidelines for adopting digital biomarkers, including:

- **Clinical relevance:** Digital biomarkers must be rigorously validated against gold-standard methodologies, including HbA1c testing, continuous glucose monitoring (CGM), and self-monitoring of blood glucose (SMBG), to ensure their accuracy and reliability.
- **Stakeholder engagement:** Early-stage collaboration involving patients, healthcare providers, and industry stakeholders is critical to ensuring practicality, accuracy, and real-world applicability of noninvasive glucose monitoring technologies.
- **Regulatory integration:** Engaging regulatory bodies at the early stages of development will streamline the approval process and support the seamless integration of wearable glucose biosensors into clinical practice, further establishing their clinical utility in diabetes care.

### Regulations and Standards

Wearable glucose biosensors fall within the category of in vitro diagnostic medical devices (IVDs) as defined by Regulation (EU) 2017/746 (IVDR). These devices continuously measure glucose levels in biofluids such as sweat, interstitial fluid, or saliva, providing vital health data essential for effective diabetes management. Achieving compliance with international regulatory standards is crucial for gaining market approval and ensuring patient safety. Important regulatory standards encompass ISO 20916, addressing clinical performance studies specifically tailored to digital glucose biosensors; ISO 14971, outlining risk management protocols designed for wearable medical devices; ISO 62304, governing software lifecycle management for AI-driven biosensor systems; and ISO 13485, ensuring that design, development, and manufacturing processes for medical wearables are appropriately aligned. Additionally, AI-powered glucose monitoring systems must adhere to the US FDA Software as a Medical Device (SaMD) regulations, which emphasize transparency through strict adherence to pre-specifications and defined algorithm change protocols.

In the European Union, compliance with the General Data Protection Regulation (GDPR) mandates clear patient communication regarding automated decision-making processes involved in AI-powered medical wearables. Similarly, the US FDA requires comprehensive documentation on data management, algorithm adjustments, and clinical validation to guarantee patient safety and product reliability. Given the continuous evolution of AI algorithms, ongoing compliance with regulatory guidelines for SaMD is critical to streamline approval processes and maintain public trust.

The commercial viability of wearable glucose biosensors developed by industry leaders like Abbott, Medtronic, and Dexcom relies on several key factors. Efficient manufacturing processes ensuring scalability and affordability are essential for broader market accessibility. Adherence to stringent international regulatory standards and certification requirements ensures product safety and effectiveness. Successful navigation of a highly competitive market landscape demands continuous technological innovation and proven reliability. Furthermore, achieving interoperability with existing medical devices, healthcare platforms, and digital ecosystems enhances user convenience, clinical utility, and overall market adoption.
